# Transition from unclassified *Ktedonobacterales* to *Actinobacteria* during amorphous silica precipitation in a quartzite cave environment

**DOI:** 10.1038/s41598-021-83416-5

**Published:** 2021-02-16

**Authors:** D. Ghezzi, F. Sauro, A. Columbu, C. Carbone, P.-Y. Hong, F. Vergara, J. De Waele, M. Cappelletti

**Affiliations:** 1 Department of Pharmacy and Biotechnology, University of Bologna, 40126 Bologna, Italy; 2 Laboratory of NanoBiotechnology, IRCCS Istituto Ortopedico Rizzoli, 40136 Bologna, Italy; 3 Department of Biological Geological and Environmental Sciences, University of Bologna, 40126 Bologna, Italy; 4 La Venta Geographic Explorations Association, 31100 Treviso, Italy; 5 Teraphosa Exploring Team, Puerto Ordaz, Venezuela; 6 Department of Earth, Environment and Life, University of Genoa, 16132 Genoa, Italy; 7 Division of Biological and Environmental Science and Engineering, King Abdullah University of Science and Technology (KAUST), Thuwal, 23955-6900 Saudi Arabia

**Keywords:** Biodiversity, Environmental microbiology, Geomorphology

## Abstract

The orthoquartzite Imawarì Yeuta cave hosts exceptional silica speleothems and represents a unique model system to study the geomicrobiology associated to silica amorphization processes under aphotic and stable physical–chemical conditions. In this study, three consecutive evolution steps in the formation of a peculiar blackish coralloid silica speleothem were studied using a combination of morphological, mineralogical/elemental and microbiological analyses. Microbial communities were characterized using Illumina sequencing of 16S rRNA gene and clone library analysis of carbon monoxide dehydrogenase (*coxL*) and hydrogenase (*hypD*) genes involved in atmospheric trace gases utilization. The first stage of the silica amorphization process was dominated by members of a still undescribed microbial lineage belonging to the *Ktedonobacterales* order, probably involved in the pioneering colonization of quartzitic environments. *Actinobacteria* of the *Pseudonocardiaceae* and *Acidothermaceae* families dominated the intermediate amorphous silica speleothem and the final coralloid silica speleothem, respectively. The atmospheric trace gases oxidizers mostly corresponded to the main bacterial taxa present in each speleothem stage. These results provide novel understanding of the microbial community structure accompanying amorphization processes and of *coxL* and *hypD* gene expression possibly driving atmospheric trace gases metabolism in dark oligotrophic caves.

## Introduction

Silicon is one of the most abundant elements in the Earth’s crust and can be broadly found in the form of silicates, aluminosilicates and silicon dioxide (e.g., quartz, amorphous silica). The knowledge of the processes involved in silica mobilization and precipitation in colloidal forms (amorphization) is of great interest for the comprehension of the formation of ancient natural silica-rich environments. Additionally, amorphous silica structures are good candidates as biosignatures for the investigation of life forms on other planets, because they represent potential analogues with silica deposits detected on Mars^1–5^.

Up to date, the precipitation of silica leading to the formation of silica sinters has been mainly described in geothermal springs and deep-sea hydrothermal fields, as a process dependent on abiotic physical and chemical factors, in which microbes play a secondary role^6–8^. On the other hand, silica mobilization and precipitation processes are believed to be mediated by biological activities in physicochemically stable environments^9,10^. In the last twenty years, microbial diversity was studied in this type of environments such as quartz substrates at a high-altitude tundra location, hyper-arid deserts, Antarctic soils and orthoquartzitic caves^9–14^. Among these, the orthoquartzitic Imawarì Yeuta cave represents a non-thermal and mild environment, which hosts a wide variety of unique amorphous silica deposits^10^. This cave is also one of the oldest caves discovered so far (20–30 Ma old) and is considered a pristine natural place, which is hardly accessible by humans^15,16^. In addition to the absence of light, some areas of the cave are isolated from the external surface, with limited air and water exchanges, determining a general low nutrient availability and low organic carbon sources. In the first work on Imawarì Yeuta cave^10^, a reciprocal effect between complex chemotrophic bacterial communities and silica-rich environments was indicated to support specific speleothem development and increasing amorphization levels in different cave niches. A high portion of the microbial communities described in the five Imawarì Yeuta speleothem samples, included in this first work, was shown to belong to *Proteobacteria*, *Actinobacteria* and *Acidobacteria*. Members of *Planctomycetes*, *Chloroflexi* and Candidate Division WPS-2 were also present in all these samples but with a lower abundance (< 3%). Interestingly, a high portion (> 70%) of the sequences obtained from the five Imawarì Yeuta samples resulted unclassified at family and genus level, suggesting the peculiarity of the microbial diversity thriving in Imawarì Yeuta cave^10^.

In oligotrophic environments that are aphotic (i.e., lacking photosynthetic organisms) and characterized by interstitial water sources with extremely low carbon content^17^, the capacity to scavenge the atmospheric trace gases H_2_ and CO might contribute to primary productivity and microbial life colonization and development^18^. Specifically, genes encoding the type I carbon monoxide dehydrogenase (CODH encoded by *cox* genes) and the [NiFe]-hydrogenase (encoded by *hyp* genes) have been correlated to the bacterial capability to utilize H_2_ and CO as energy source for cell growth and persistence under nutrient-limiting conditions^19^. Up to date, members of the only three phyla *Chloroflexi*, *Actinobacteria* and *Acidobacteria* have been functionally associated with CO and H_2_ consumption/uptake of ecological and biogeochemical interest^19^. By targeting *cox* and *hyp* genes in microbial community characterization studies^18–21^, trace gas oxidizers members of *Chloroflexi*, *Actinobacteria* or *Acidobacteria* resulted to be predominant in oligotrophic ecosystems, not only of volcanic origin and geothermal sites^18^.

In the present work, we combined geochemical, morphological, and microbiological analyses to characterize three speleothems representing consecutive silica amorphization steps leading to coralloid silica formation in Imawarì Yeuta Cave (Venezuela). Silica coralloid speleothems have been widely documented in silica-rich environments and their origin was suggested to be associated to microbial activities, mostly on the basis of geomorphological observations^22–24^. In order to delve deep this aspect, we have conducted microbiological analyses using Illumina sequencing targeting the 16S rRNA gene (both hypervariable regions and near-full length sequences) and clone library analysis of genes encoding the type I carbon monoxide dehydrogenase (*coxL*) and a maturation factor of the [NiFe]-hydrogenase (*hypD*), which are known to mediate aerobic respiration of atmospheric gases and might contribute to microbial growth under oligotrophic conditions^18,19^.

## Results

### Speleothems description

**Figure 1:**
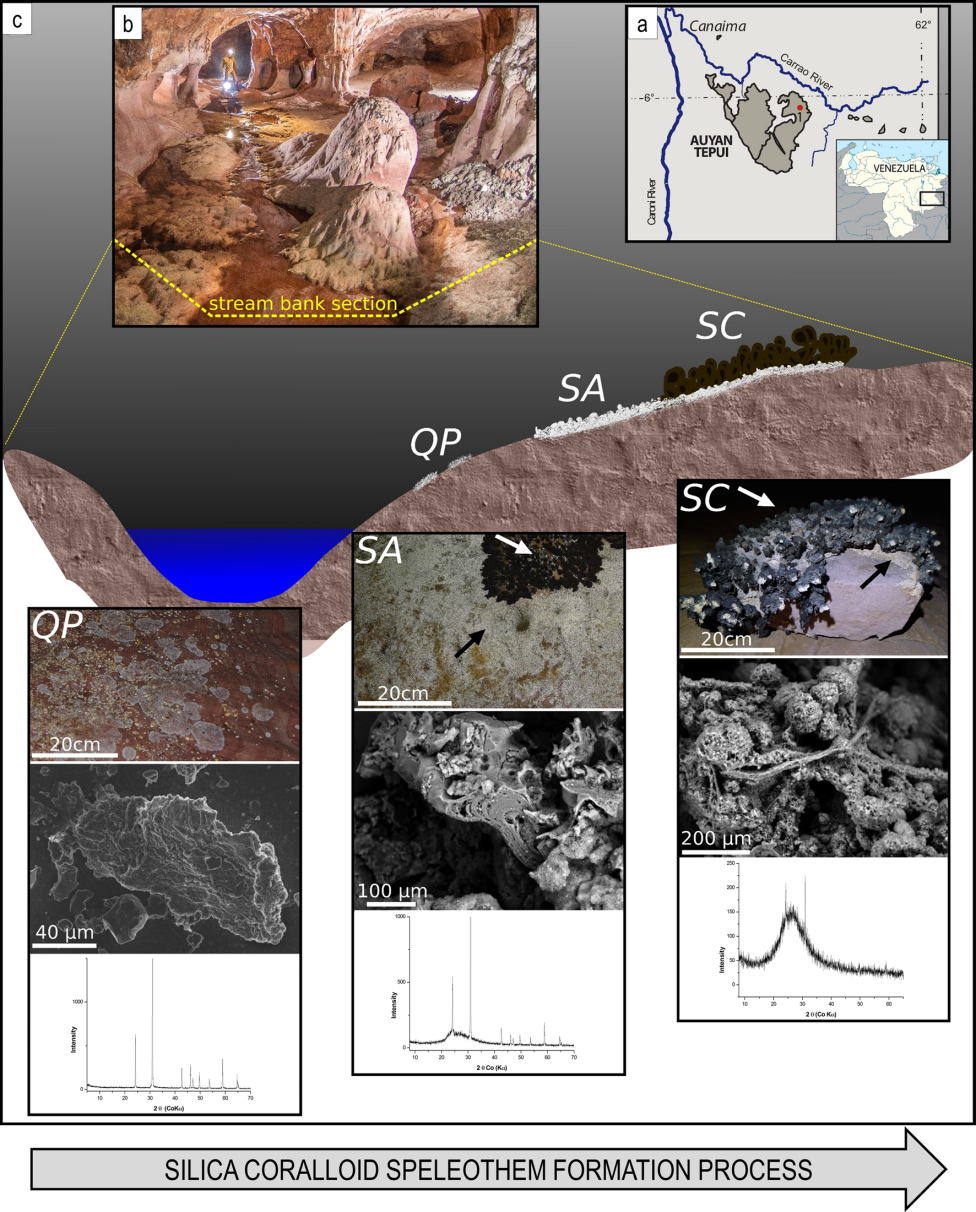
Study area, cave system and samples. (**a**) Geographical map indicating the location of Auyan Tepui table-mountain in Venezuela. (**b**) Picture of the cave location where the samples were collected along a stream bed with the indication of the stream bank section that is enlarged and schematized in panel C. (**c**) Schematic representation of the location and distance/height of samples QP, SA and SC from the stream bed. Black and white arrows point to SA and SC respectively. For each of the sample, the morphology, the FESEM image and the XRD pattern are shown. Photos are provided by La Venta Archive.

We analysed three different but proximal portions of the floor surface showing different visible aspects with respect to quartzite bedrock. They were collected on the cave floor of a gallery traversed by a small stream fed by waters percolating through rock fractures (measured discharge: 0.1 L s^−1^) in the inner part of Imawarì Yeuta cave (Auyan Tepui, Venezuela). The pH of this water was 5, which was higher than that measured in the main cave streams (between 3 and 4.5)^17^ and similar to that measured in standing pools of water with dissolved silica close to saturation in the same area of the cave (pH between 5 and 5.5). These infiltration/interstitial waters are transparent and show extremely low conductivity values (close to distilled water) with contents of organic acids and dissolved carbon always below the detection limits^17^. On the basis of the relative proximity and appearance, the three portions of the floor surface were identified as consecutive stages of silica amorphization process leading to the silica coralloid speleothem (QP, pristine quartz; SA, amorphous silica; SC, coralloid silica) (Fig. 1). The portion QP was a patina/biofilm covering the pristine quartzite rock and was collected at less than 1 m of distance and 15 cm in height from the stream bed (Supplementary Fig. S1). QP had the appearance of greyish patina in the form of centimetric patches on the rock surface with a moist texture (Fig. 1). Moving further and higher from sample QP along the orthoquartzite floor, there was a transition from the greyish patches to a whitish and soft paste of amorphous silica distributed in closely arranged dots that in places merge to form a continuous layer covering the floor. Some of these dots were collected, as representative of sample SA. Further, immediately above these dots, blackish and hard branched formations—like cave coralloids^25^—of amorphous silica appeared as compact dry masses that were collected and named SC (Fig. 1).

### Geochemical and micro-fabric analyses

QP was almost exclusively constituted by quartz grains, while SA and SC appeared as gel-like and hard amorphous silica speleothems, respectively (Fig. 1). Mineralogical and geochemical analyses showed that the two amorphous silica samples were mainly constituted of Opal-A (SiO_2_·*n*H_2_0) (Fig. 1, Supplementary Fig. S2). Aside of silica, major elements homogeneously detected with XRF analysis across the three samples were Al and Fe. Trace elements, metals, and anions like Ba, SO_4_, Cu, Zn and Cl increased in abundance going from QP to SA and SC (Supplementary Fig. S1).

The observation of the three siliceous formations with FESEM revealed the presence of microbial-like morphologies, although in different abundance and density. QP quartz grains were covered by sparse long filamentous biological structures that were directly adhered on quartz-based substrates. The microbial colonisation and the complexity of the biological structures increased in samples SA and SC. The first showed irregular distribution of cotton-shaped extracellular deposits and/or matrix mixed up with silicified tubular casts and peloids. The SC samples were composed of tiny coralloid bodies. In this speleothem, tubular sheets (Fig. 1, Supplementary Fig. S2), filaments and spore-like chains constituted a compact aggregate in which amorphous silica consolidates the chains forming intertwined structures. The enlarged alveolar surface visible on the SC surface could correspond to *Actinobacteria*-like hyphae, thin spider threads, or a combination of both (Fig. 1, Supplementary Fig. S2).

### Microbial diversity characterization

#### Abundance of bacterial and archaeal strains

Bacterial and archaeal 16S rRNA genes were analyzed with qPCR in the three speleothems. SA showed the highest number of bacterial 16S rRNA copies per gram of raw sample (3.92 × 10^9^), followed by QP (1.39 × 10^8^) and SC (7.57 × 10^5^). Archaeal 16S rRNA copies were three orders of magnitude lower than the bacterial ones in all speleothems (2.53 × 10^5^ in QP, 9.5 × 10^6^ in SA and 8.48 × 10^2^ in SC).

#### Statistical analyses and diversity indexes

Among the three samples, QP showed the highest richness, while sample SC had the highest diversity in terms of Shannon and Simpson’s indexes (Table S2). The Pielou’s index also indicated an increase of evenness moving from QP sample to SA and SC. This suggests a lower number of dominant species in the amorphous and coralloid silica samples as compared to the quartzite (non-amorphous silica) speleothems. Further, by considering the taxonomy analysis, the first two speleothems clustered together and separately from QP, indicating a higher similarity in microbial community composition in SA and SC (Supplementary Fig. S3).

For each speleothem, the taxonomy classification of the Amplicon Sequence Variants (ASVs) was compared with that of the bacterial and the archaeal Operational Taxonomic Units (OTUs) in order to assess the robustness of the data describing the microbial communities’ composition. The Pearson correlation value between ASV and bacterial OTU dataset was high enough in all speleothems (ρ > 0.7) to indicate that there was a significant correspondence in the microbial characterization described through the analyses of the V4–V5 region and the near-full length bacterial 16S rRNA gene. In particular, the maximum correlation value between ASVs and bacterial OTUs was obtained for the QP sample (ρ = 0.96) (Supplementary Fig. S3). On the other hand, a ρ < 0.6 was found between ASV and archaeal OTU in all samples (data not shown), indicating that the archaeal primers did not represent the microbial communities defined by the V4–V5-targeting primers that are known to cover both archaeal and bacterial diversity present in various microbial communities^26^.

#### Members of *Ktedonobacterales* order dominate the microbial community in the pristine quartzite surface

**Figure 2:**
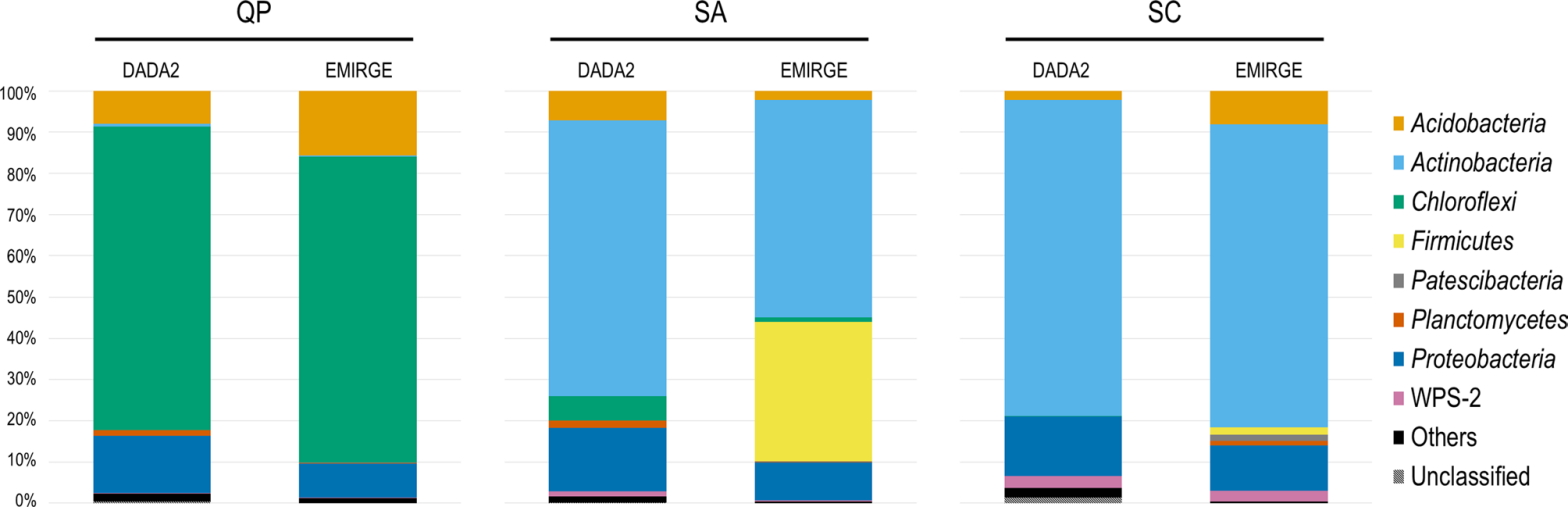
Distribution of microbial phyla in the three cave samples based on Amplicon Sequence Variants (ASVs, named DADA2) and bacterial Operational Taxonomic Units (OTUs, named EMIRGE). “Others” represents microbial phyla that constitute less than 1% in all samples.

By using default parameters (minimum identity parameter of 0.95) for taxonomy assignment in SILVA database, the three 16S rRNA sequences datasets describing the QP microbial community resulted to be dominated (> 70% of the entire community) by unclassified bacteria. Only by reducing the minimum identity parameter to 0.8, these unclassified sequences were all affiliated to *Chloroflexi* phylum of *Ktedonobacteria* class and *Ktedonobacterales* order (Fig. 2).

**Figure 3:**
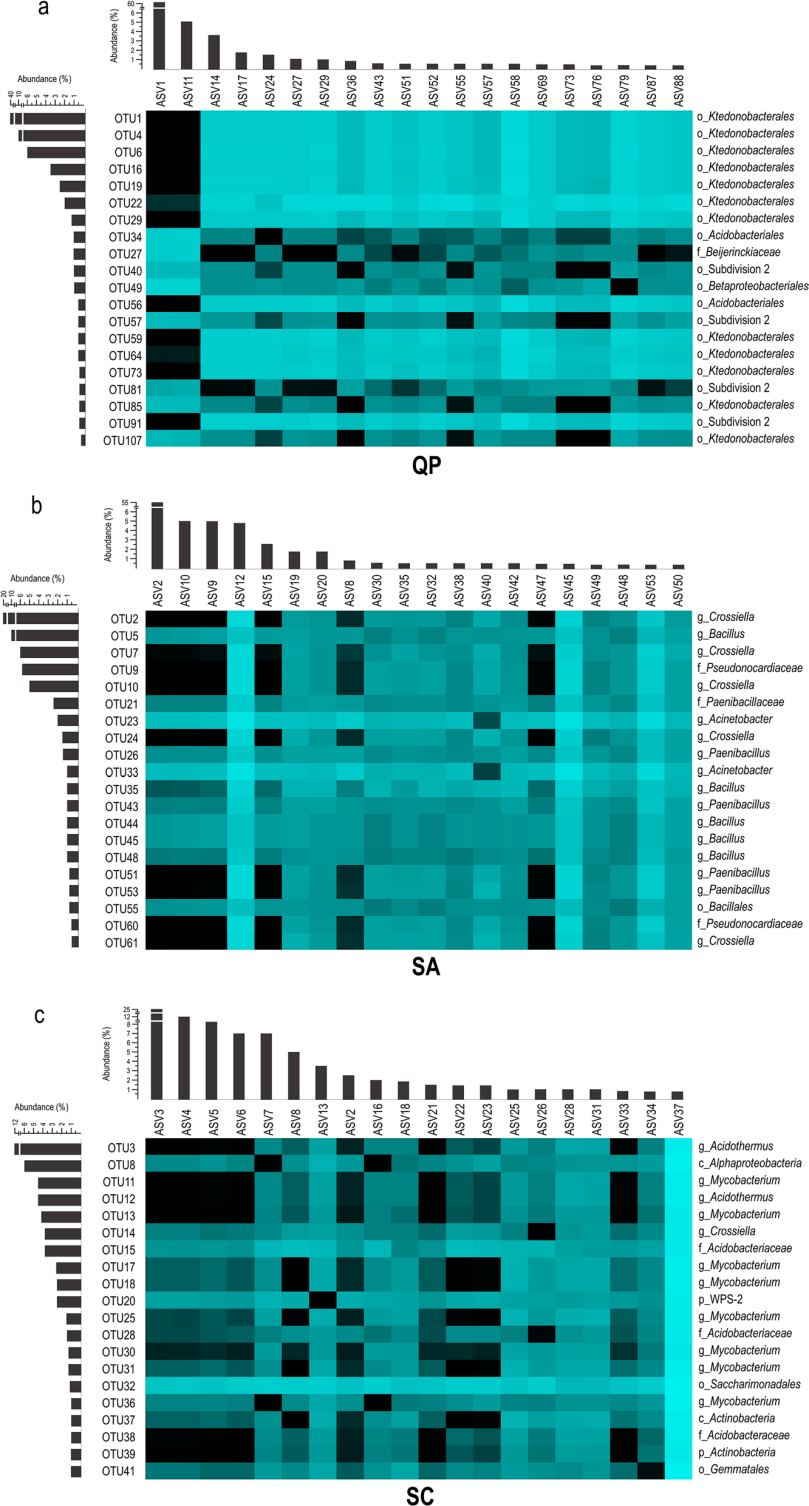
Heat maps showing the nucleotide similarity among the 20 most abundant ASVs and OTUs found in QP (**A**), SA (**B**) and SC (**C**) microbial communities. The green graduation levels refer to a minimum value of 50% and a maximum value of 100% of nucleotide identity. The bars in grey show the relative abundance of the reported ASV and OTU within each community. The SILVA-based taxonomy affiliation of the OTUs is indicated as follows, p = phylum, c = class, o = order, f = family, g = genus. Primer-E v7 software (www.primer-e.comwww.primer-e.com) was used to create the heat maps showing the percent nucleotide similarity between ASVs and OTUs that was calculated through multiple sequence alignments with Clustal Omega (https://www.ebi.ac.uk/Tools/msa/clustalo/).

In the ASV dataset, almost the entire *Ktedonobacterales* community was constituted only by two ASVs, which had a high similarity (> 98%) among each other. These two ASVs found correspondence (> 97% similarity) with the six most abundant bacterial OTUs and the four *Ktedonobacterales*-related sequences retrieved from the clone library (Fig. 3A). Other abundant phyla in QP were *Proteobacteria* and *Acidobacteria* (6–20% abundance in ASV and bacterial OTU datasets) (Fig. 2). Proteobacterial members mainly belonged to *Alphaproteobacteria* of *Rhizobiales* and *Esterales* orders, and to *Gammaproteobacteria* of *Betaproteobacteriales* (Supplementary Fig. S4). Among these, *Bejerinckiaceae* and *Burkholderiaceae* families were the most abundant families among *Proteobacteria*, while *Acidobacteria* were mainly represented by Subdivision 2 and *Acidobacteriales* orders, unclassified at lower taxonomy levels (Supplementary Fig. S5).

**Figure 4:**
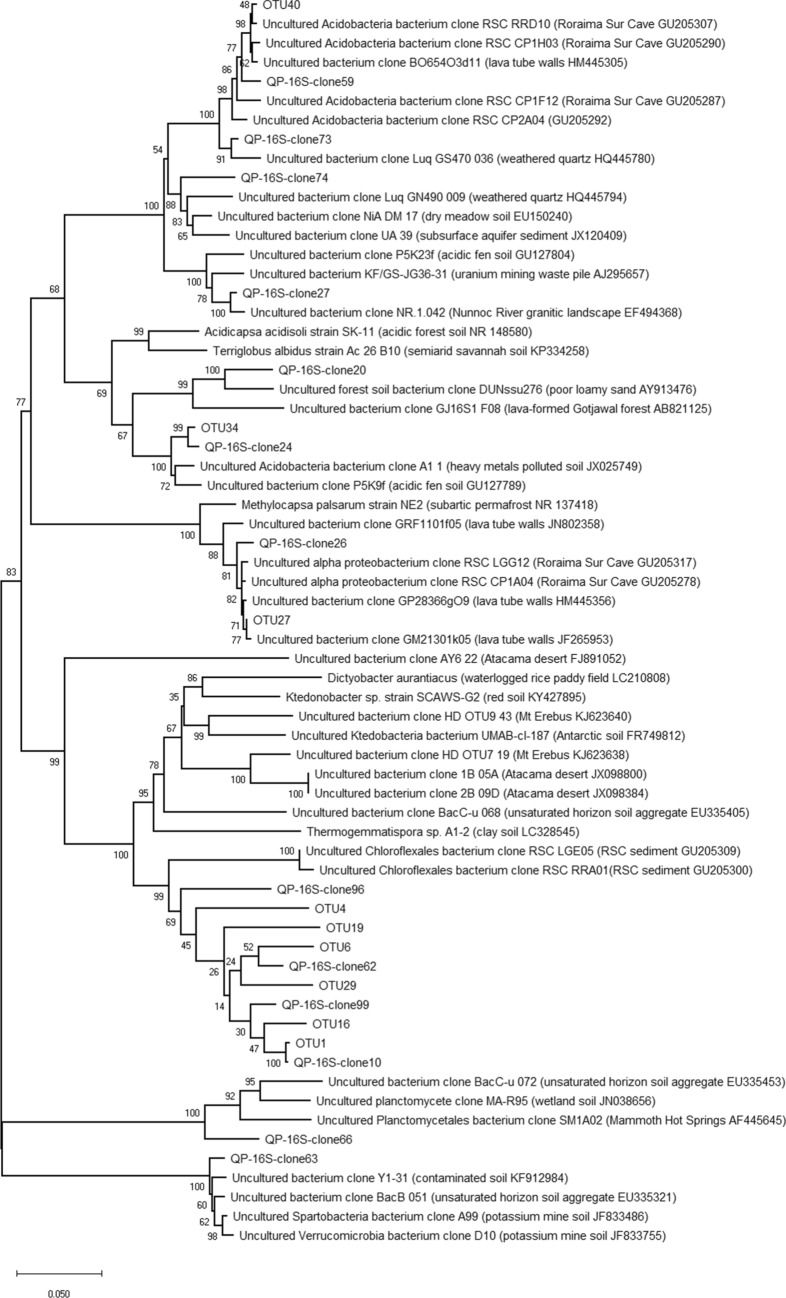
Neighbour-joining tree showing the phylogenetic affiliation of the dominant bacterial OTUs (named “OTU#”) and the 16S rRNA clone group sequences (“named QP-16S-clone#”) in the sample QP. Bootstrap values (based on 1000 replications) are shown at branch points. Bar, 0.05 substitutions per nucleotide position.

In the phylogenetic analysis including the near-full length 16S rRNA sequences of the QP bacterial OTUs (retrieved from Illumina and EMIRGE analysis) and clone library sequences (retrieved from Sanger sequencing of representative clones), the *Ktedonobacterales*-related sequences had limited sequence similarity (< 90%) with other sequences present in the database. Among these, they showed the maximum similarity value with clone sequences retrieved from the only other orthoquartzitic cave microbiologically described up to date, i.e., Roraima Sur Cave^9^. These sequence identity values decreased (71–87%) by comparing the QP 16S rRNA sequences with clone sequences affiliated to *Ktedonobacterales* retrieved from other quartz-rich environments, i.e., cold, arid and nutrient poor soils^9–11,13,14^ (Fig. 4, Table 1). The most abundant proteobacterial and acidobacterial sequences shared > 97% of sequence identity with clone sequences of uncultured microorganisms retrieved from Roraima Sur Cave, lava tube walls, and other volcanic deposits featured by biological CO-oxidizing activities (Fig. 4). Low abundant bacterial phyla that were detected in all the 16S rRNA sequence datasets were *Planctomycetes* and *Verrucomicrobia*.

**Table 1:** Comparison between the QP *Ktedonobacterales* 16S rRNA clone sequences, the ASV and OTU sequences from the Imawarì Yeuta samples and the *Chloroflexi*-related sequences retrieved from other quartzite environments.

Imawarì Yeuta												Roraima Sur Cave^9^	Atacama Desert^12^	Mt Erebus^14^
QP^b^				SA^b^			SC^b^			Sample Q^10^	Sample S^10^			
Clone^a^	ASV	Bacterial OTU	Archaeal OTU	ASV	Bacterial OTU	Archaeal OTU	ASV	Bacterial OTU	Archaeal OTU					
QP-16S-clone10	100	100	96.9	77.8	83.2	82.1	76.3	–^c^	–	88.3	86.0	90.6	84.0	85.1
QP-16S-clone62	100	94.4	88.7	77.8	80.4	79.5	76.3	–	–	88.3	86.0	86.9	81.7	80.8
QP-16S-clone96	100	90.5	96.8	77.8	83.4	82.1	76.3	–	–	88.3	86.0	86.0	81.6	85.2
QP-16S-clone99	100	93.7	96.8	77.8	83.4	82.5	76.3	–	–	88.3	86.0	87.8	82.5	84.2

Nucleotide sequence identity (expressed in %) was calculated by using Blast algorithm.

Numbers in apex correspond to the references.

^a^Representative clones from the 16S rRNA clone library of QP that were Sanger sequenced.

^b^Sequencing data obtained in this work.

^c^“–” indicates the absence of OTUs affiliated to *Chloroflexi* in the corresponding dataset.

The archaeal OTU dataset was used to provide taxonomy information on the archaeal sequences present in QP. Around 60% of the archaeal OTU dataset was composed by sequences almost exclusively belonging to *Thaumarchaeota* phylum of *Nitrososphaeria* and *Group 1.1c* classes and unclassified at lower taxonomy levels, while *Crenarchaeota* were < 2% (Supplementary Fig. S6). Despite the specificity of these primers towards archaea, a high abundance (26%) of *Ktedonobacterales*-related sequences was also revealed in the archaeal OTU dataset (data not shown), probably because of the strong dominance of this bacterial order in the QP microbial community. On the other hand, no archaeal sequences were retrieved in the bacterial OTU dataset and < 0.5% of sequences belonged to this domain within the ASV dataset (in “Others” in Fig. 2). These results were taken as evidences of the low abundance of *Archaea* in QP. The phylogenetic analysis of the *Thaumarchaeota*-affiliated sequences showed their highest similarities with clones retrieved from Roraima Sur Cave and from environments featured by ammonia-oxidizing processes and acidic pH values (Supplementary Fig. S7).

#### Different acidophilic *Actinobacteria* groups prevail during the amorphization progression towards the formation of coralloid silica speleothems

Moving towards more advanced stages of the silica amorphization process, the microbial community composition shifted towards the dominance of *Actinobacteria* (50–70% in SA, 70–80% in SC) that was accompanied by a gradual decrease of *Chloroflexi* (< 6% in SA and < 0.1% in SC) (Supplementary Fig. S3). In particular, the *Actinobacteria* phylum was mainly composed of members of the genus *Crossiella* of *Pseudonocardiales* order in SA and by members of the genus *Acidothermus* of *Frankiales* order in SC, respectively. *Mycobacterium* genus of *Corynebacteriales* order was also detected in both samples, although it was highly abundant in SC only (Fig. 3b,c, Supplementary Fig. S4). Generally, during the progression towards amorphous and coralloid silica states, taxa with very low abundance in the quartzitic sample QP increased. Indeed, members of these *Actinobacteria* groups were also present in QP sample but they were in traces (< 0.5%) (Fig. 2). The only EMIRGE approach also revealed the high abundance (34%) of members of *Firmicutes* phylum within the SA microbial community (Fig. 2), mainly represented by *Bacillus* and *Paenibacillus* genera (Fig. 3b,c). The lack of sequences in the ASV dataset belonging to this phylum might be due to the different primer set used^27,28^.

Additional abundant bacterial phyla detected in the microbial communities of SA and SC using both the approaches were *Proteobacteria* (9–15% in SA, 11–14% in SC), and *Acidobacteria* (2–7% in SA, 2–8% in SC) (Fig. 2). At lower taxonomy level, *Proteobacteria* in SA and SC communities were mainly composed of *Alphaproteobacteria* of *Acetobacteraceae* family and *Gammaproteobacteria*, mainly belonging to *Acinetobacter* genus in SA and to *Burkholderiaceae* family in SC. Conversely, *Acidobacteria* mainly included members of *Acidobacteriales* in SC, and members of both *Acidobacteriales* and Subdivision 13 in SA (Fig. 3b,c, Supplementary Figs. S4, Supplementary Fig. S5). Additional phyla present (< 3%) in both SA and SC were *Planctomycetes* and WPS-2. *Patescibacteria* were > 1% only in SC and only within the OTU dataset (Fig. 2).

**Figure 5:**
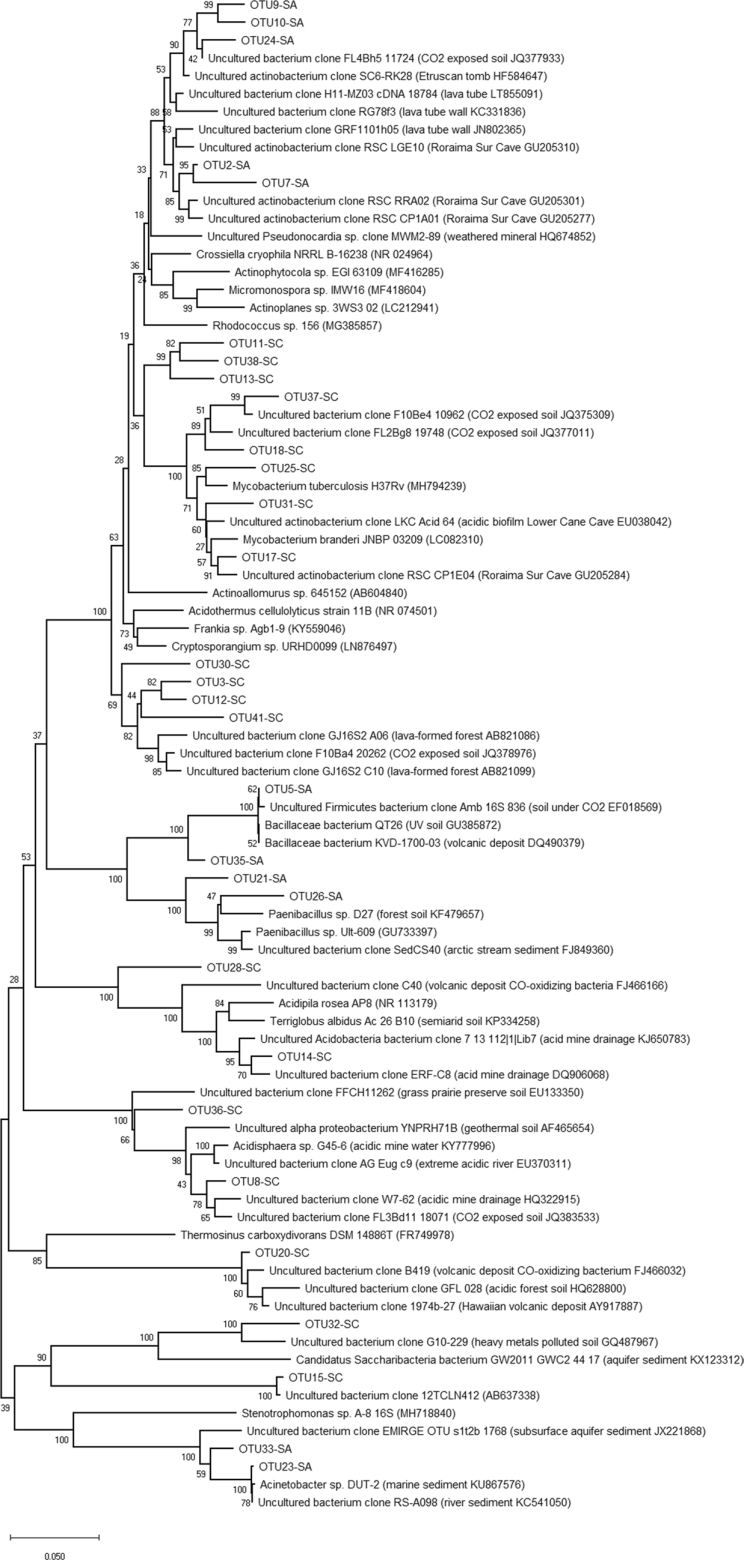
Neighbour-joining tree showing the phylogenetic affiliation of the dominant bacterial OTUs (named “OTU#”) in the samples SA and SC. Bootstrap values (based on 1000 replications) are shown at branch points. Bar, 0.05 substitutions per nucleotide position.

In the phylogenetic tree, the dominant *Crossiella*-related OTUs clustered in two different clades. One clade was highly related (> 98%) to clones retrieved from Roraima Sur Cave, while the other showed affiliation (> 98%) with clones retrieved from soil exposed to high CO_2_ concentration (Fig. 5). Further, the *Bacillus*-related OTU5 abundant (9.1%) in SA community EMIRGE dataset showed high similarity (99%) with sequences from volcano deposits and soils exposed to radiation or to CO_2_ (Fig. 5). On the other hand, in the only ASV dataset, a *Ktedonobacterales*-related sequence (ASV352) was abundant (4.8%) in SA. This sequence showed low similarity with ASVs from QP (maximum sequence identity of 79%) and was related (similarity of 90–92%) with clone sequences retrieved from acidic sulphate soil and heavy metal-contaminated environments^29,30^.

In SC microbial community, the dominant *Acidothermus*-related sequences formed one clade with a maximum of 96% similarity with reference sequences of acidophilic actinomycetes retrieved from acid environments (Fig. 5). Similarly, one abundant (> 5%) *Alphaproteobacteria*-related sequence was present in each dataset (ASV120 and OTU8), which was affiliated (> 96% similarity) with clone sequences retrieved from subterranean acidic environments (Fig. 5).

As revealed in QP microbial community, archaeal sequences represented 0.6% of the entire population in SA. The archaeal OTU datasets indicated that moving from QP to SA, there was an enrichment of *Crenarchaeota*, mainly of *Bathyarchaeia* class, in the archaeal community (Supplementary Fig. S6). *Thaumarchaeota* were detected at 13% in SA archaeal OTU dataset and were totally composed of members belonging to the *Nitrososphaeira* class. On the other hand, no archaeal sequences were retrieved from SC archaeal OTU dataset. In the phylogenetic tree, they clustered with sequences collected from acidic hot springs (Supplementary Fig. S7).

### Diversity of bacterial *coxL* and *hypD* genes in the consecutive stages of silica amorphization

**Figure 6:**
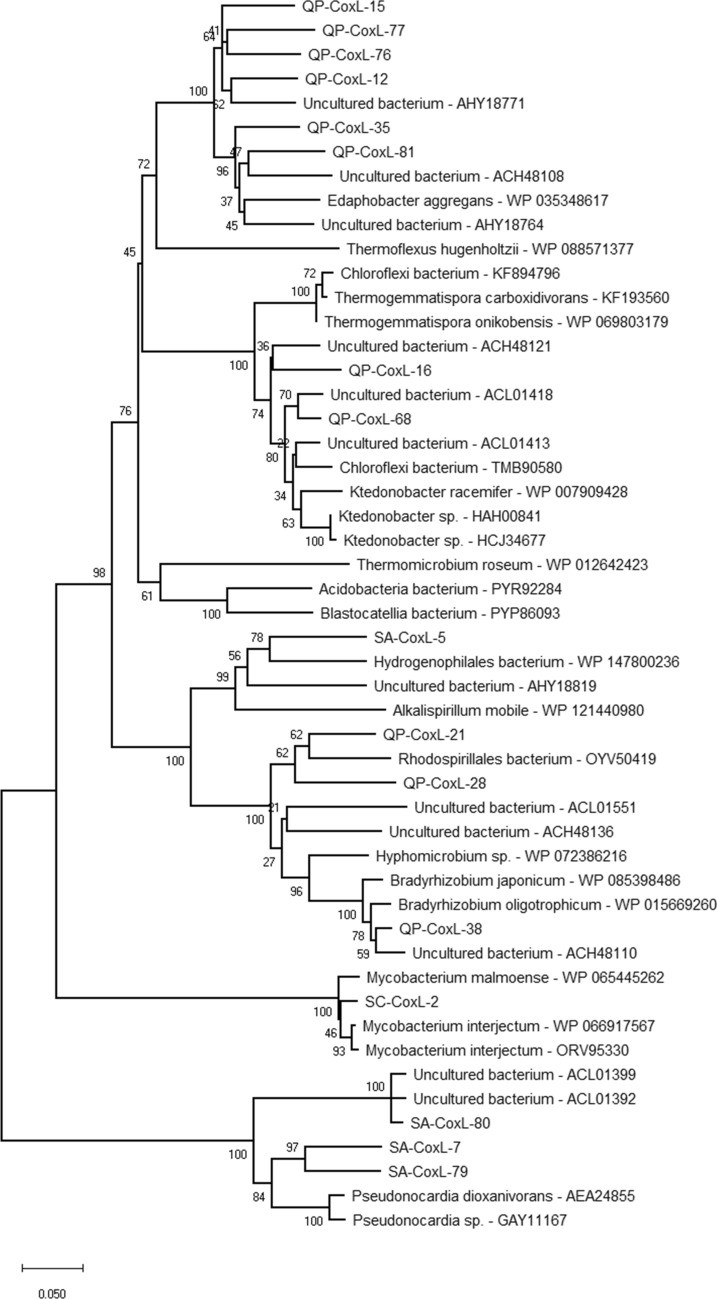
Neighbour-joining tree showing the phylogenetic affiliation of the CoxL clone group sequences from the samples QP, SA and SC (named “QP/SA/SC-CoxL-#”). Bootstrap values (based on 1000 replications) are shown at branch points. Bar, 0.05 substitutions per amino acid position.

The diversity of both CoxL and HypD sequences was higher in QP as compared to SA and SC (Table S4). The RFLP-based screening of the *coxL* clone library of QP showed the presence of 11 groups. Around half of the clone library showed affiliation with CoxL reference sequences from members of different genera of *Chloroflexi* phylum and from one *Edaphobacter aggregans* strain (Fig. 6, Table S5). In particular, the *Edaphobacter*-related CoxL sequences clustered in two groups; one of these represented the most abundant group in the QP CoxL clone library (QP-CoxL-clone76 in Table S5) and did not include any close reference sequences. Interestingly, although *Edaphobacter* belongs to the *Acidobacteria* phylum, it clustered with CoxL sequences of *Chloroflexi* and separately from those of other *Acidobacteria* genera. The rest of the QP CoxL library was represented by sequences related to members of *Rhizobiales* and *Rhodospirillales* of *Alphaproteobacteria* (Fig. 6, Table S5). Conversely, the *coxL* clone library from SA sample was dominated by sequences belonging to *Actinobacteria*, mainly related with *Pseudonocardia* genus, while 30% of the sequences were affiliated with *Betaproteobacterales* (Fig. 6, Table S5). The CoxL clone library of SC were completely represented by *Mycobacterium*-related sequences (Table S5). In the phylogenetic tree, most of the CoxL reference sequences affiliated with clones from QP and SA libraries shared their highest sequence similarity with clones retrieved from volcanic deposits in Hawai’i and Japan^20,31^ (Fig. 6).

**Figure 7:**
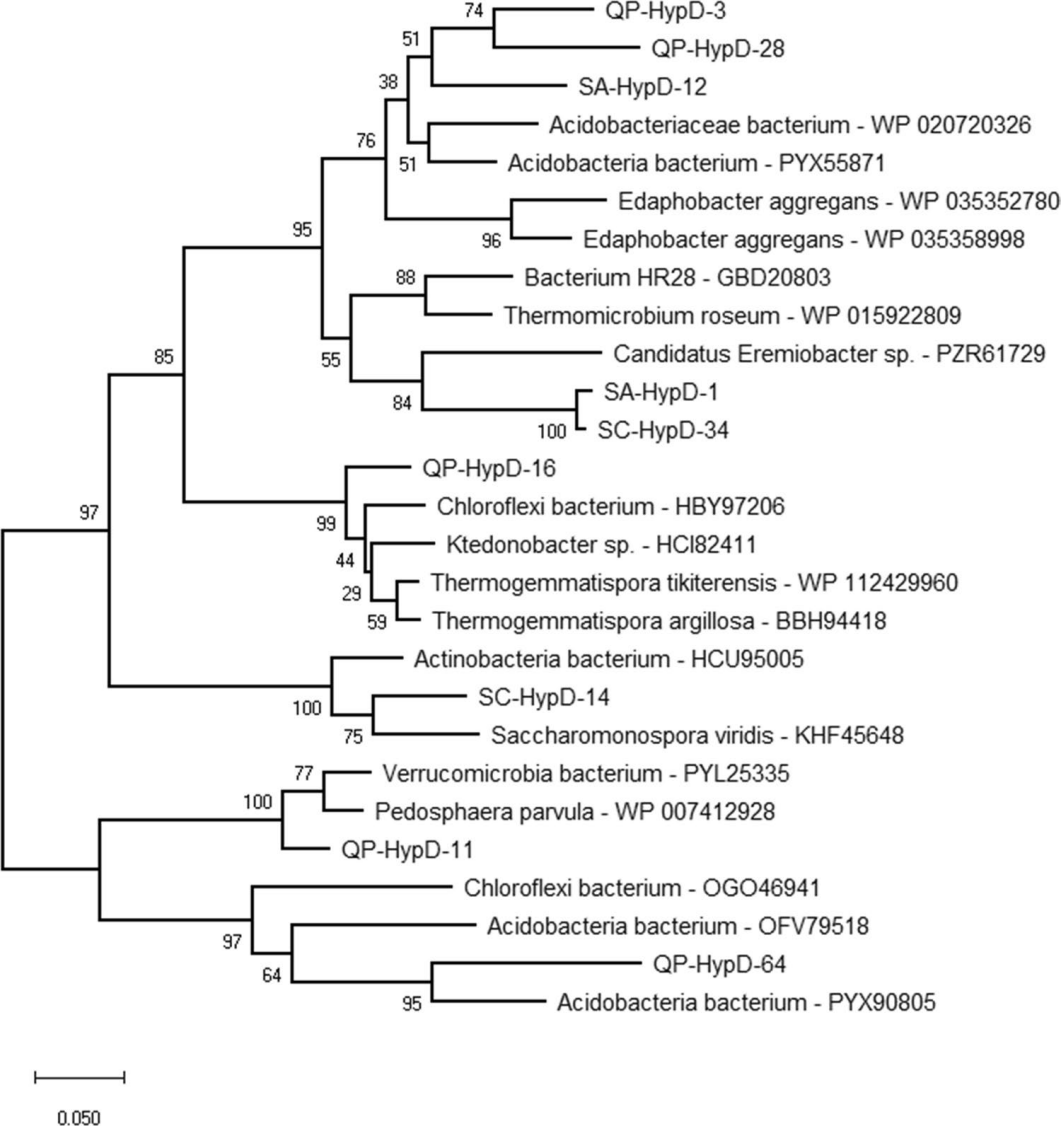
Neighbour-joining tree showing the phylogenetic affiliation of the HypD clone group sequences from the samples QP, SA and SC (named “QP/SA/SC-HypD-#”). Bootstrap values (based on 1000 replications) are shown at branch points. Bar, 0.05 substitutions per amino acid position.

The analysis of *hypD* clone libraries showed a dominance of *Acidobacteria*-related HypD sequences in QP, while the rest of the library showed affiliation with members of *Chloroflexi* and *Verrucomicrobia* phyla (Fig. 7, Table S6). The HypD clone library of SA was almost equally distributed between clones affiliated with *Acidobacteria* phylum and clones related with WPS-2 phylum (recently proposed as *Candidatus* Eremiobacteraeota^18^) from Antarctic desert soils (Table S6). In SC sample, one single HypD clone group represented the entire library which was affiliated with *Actinobacteria Saccharomonospora* retrieved from a fossil Centrosaurus bone from the Late Cretaceous^32^. In the phylogenetic tree, the *Acidobacteria*-like HypD sequences from SA clustered together with those from QP and separately from the reference sequences, indicating their limited representation in the database. While *Chloroflexi*- and *Verrucomicrobia*-related sequences revealed similarities with clones retrieved from meadow soil, phylogenetic analysis of *Ktedonobacteria*-like sequences were related to clones retrieved from clay soil in geothermal areas (Fig. 7).

## Discussion

A large diversity of silica coralloid speleothems was reported in different cave systems and an increasing number of observations indicated them as representative of proper stromatolites^33,34^. This work describes the microbial communities characterizing the first surface colonization and the sequential silica amorphization steps during the formation of a peculiar silica coralloid speleothem collected from Imawarì Yeuta cave. The microbial community diversity and structure were assessed using Illumina sequencing approaches targeting not only the V4–V5 hypervariable regions, but also the near full-length sequences of 16S rRNA gene. The latter was done by utilizing EMIRGE method to reconstruct entire amplicons from Illumina metagenomic sequences^35^. We also performed clone library screening and Sanger sequencing in order to (1) get deep into the microbial taxonomy of one of the samples under analysis (the one possessing the highest portion of unclassified microbial population) and (2) further support the reliability of EMIRGE approach, previously reported by Miller et al.^35,36^. The sequencing of the full-length 16S rRNA gene allowed the combination of high taxonomy resolution with the depth and sensitivity provided by high-throughput sequencing data^35,36^ and increased the robustness of the microbial community analysis of the three silica amorphization stages described in this work. As a result, moving from a patina visible on the pristine orthoquartzitic surface (QP) to the amorphous silica (SA) and silica coralloid (SC) speleothems, the microbial diversity shifted from the dominance of *Chloroflexi Ktedonobacterales* to the dominance of *Actinobacteria Pseudonocardiales* and *Frankiales*, respectively. Further, the low abundant populations of *Alphaproteobacteria* and *Acidobacteria* rearranged at lower taxonomy level, showing a decrease in the abundance of alphaproteobacterial *Rhizobiales*, *Esterales* and acidobacterial Subdivision 2 accompanied by an increase of *Acetobacterales* and Subdivision 1. The enrichment and shifting of specific microbial groups during the formation of the coralloid silica speleothem was accompanied by the increase of silica amorphization level and was also associated to alkalinisation and increase of metal amount like barium and zinc. These results are in line with the parallelism between silica amorphization, metal content and pH previously reported by Sauro et al.^10^, being each of these factors both possible drivers and consequences of the microbial community composition and activity. In relation to the proximity of the Imawarì Yeuta collection site to a water stream, the water presence was an additional aspect that was suggested to influence the microbial growth and activity in the orthoquartzite Roraima Sur Cave (localized in another tepui mountain), as a possible and sporadic carbon and energy input^9^. Nonetheless, water flows in the inner zones of Tepui caves are known to be extremely oligotrophic, mildly acidic and low in dissolved minerals^16,17,37–39^. While streams on the surface of the tepuis—and when directly sinking in cave entrances—present the typical amber color due to the presence of dissolved organic acids, the water stream reported in this study is transparent since it derives from waters infiltrating through fractures which are carrying minimal amounts of organic carbon into the cave system. This is due to filtration and adsorption along the percolation pathway, as described in several other caves of the Venezuelan tepuis^16,39,40^.

The microbial community associated to the first sign of pristine orthoquartzitic surface weathering in Imawarì Yeuta (QP) was dominated by members of an undefined lineage of *Chloroflexi* phylum and *Ktedonobacterales* order. Additional 16S rRNA-based clone library analyses (associated with Sanger sequencing of the representative library clones) were also performed to support the sequences obtained through the reference-based EMIRGE method (i.e., possibly working with lower efficiency when reference 16S rRNA sequences are absent in the database). Members of *Chloroflexi* phylum are characterized by a high metabolic and genomic diversity, presenting heterotrophic, lithotrophic and phototrophic lifestyles; this phylum includes members adapted to oxic and anoxic environments and to mesophilic and thermophilic temperatures^19,41^. Despite being widespread in free-living microbial communities from different environments, the *Chloroflexi* phylum remains a relatively understudied bacterial lineage. In particular, limited knowledge is available on the diversity and ecological significance of *Ktedonobacteria* class, which includes the two orders *Thermogemmatisporales* and *Ktedonobacterales*. The few cultured strains that were characterized for this class are aerobic sporigens, with complex morphologies, actinomycetes-like mycelia and wide genome size diversity (from 5.6 to 13.7 Mbp)^42–45^. Although these strains were isolated from different types of terrestrial environments (paddy and forest soil, thermophilic compost and geothermal soils), metagenomic and 16S rRNA profiling analyses indicated the preferential *Ktedonobacteria* colonization of oligotrophic and extreme environments^46^. Among these, high abundance of *Ktedonobacteria* was previously observed in nutrient-limited, acidic environments, rich in silicate minerals that were also featured by low temperatures, like the Atacama Desert, the Antarctic Terra Nova Bay and the dark volcanic ice cave systems located in Mt. Erebus^13,14,47^, and by high CO_2_ concentration, like a gas vent in Calatrava^48^. The absence of light was also a common aspect featuring the niches colonized by members of this bacterial group. For instance, the presence of specific *Chloroflexi* members characterized the hypolithic colonization of quartzite surfaces in the Atacama Desert^12^. In this dark and extremely oligotrophic environment, the authors proposed the nitrogen and nutrient availability of undefined origin to be an important driver leading to the enrichment of this group. While the *Chloroflexi* strains were taxonomically uncharacterized in the original paper, we found their affiliation with *Ktedonobacterales* order and with the dominant taxa in QP. More in general, the *Ktedonobacterales*-related strains from QP showed a maximum similarity of 86% and 85.5% with the genera *Ktedonobacter* and *Ktedonosporobacter*, respectively*,* and a maximum similarity of 89% with members of *Ktedonobacteraceae* family. These full-length 16S rRNA sequence identities were suggested to define distinct taxa at least at the level of family^49^. However, the 16S rRNA-based analysis is often not enough for deep taxonomy studies and the only attainment of the isolate would allow the certain definition of the new bacterial lineage possibly associated to the pioneering colonization of orthoquartzite cave surfaces. In the literature, only one strain was isolated for each of the two *Ktedonobacterales* genera *Ktedonobacter*^50^ and *Ktedonosporobacter*^51^, while most of the *Ktedonobacter* genomes in the database are available as draft version and derive from metagenome-based reconstructions. Their filamentous structures resemble the microbial morphology that we observed in QP sample through FESEM, although no spore-like structures were evident in the Imawarì Yeuta sample^52,53^. By considering the similarity with the sequences present in the database, QP *Ktedonobacterales*-related full-length sequences showed the highest similarity (around 89% with a coverage of 92%) with clone sequences which resulted highly abundant in two samples collected from the orthoquartzite Roraima Sur Cave. Interestingly, the appearance and environmental conditions of QP sample resemble those described in Roraima Sur Cave that were defined as representative of ‘average’ orthoquartzite surface, which probably correspond to non-amorphous silica samples^9^. The percentage of similarity of QP *Ktedonobacterales*-related sequences decreased (similarity < 88%) when they were compared to taxonomically related sequences retrieved (in very low amount) from Imawarì Yeuta cave wall-related samples, which presented no or minimal sign of silica amorphization. The similarity further decreased (< 80%) by considering the *Ktedonobacterales*-related sequences retrieved from SA and SC, which represented more advanced silica amorphization phases and were collected in close proximity to QP^10^ (Table 1). Taken together, these results (derived from the comparison of Tepui’s orthoquartzite cave microbiology works, i.e., the present study, Barton et al.^9^, Sauro et al.^10^) suggest a high diversity among *Ktedonobacterales* members associated to orthoquartzite cave colonization, even within the same cave (Imawarì Yeuta). Their phylogenetic relationship and relative abundance seemed to be more related to the silica amorphization phase and/or pH value rather than to the relative distance among the collection sites.

In our study we found that the increase of silica amorphization and coralloid speleothem formation was accompanied with the selection of acidophilic *Actinobacteria* and, at a lower extent, *Firmicutes*. Besides the cell wall properties, the intense polysaccharide production and the complex metabolic activities were suggested to support the *Actinobacteria* role in silica solubilization and precipitation processes^54^. The presence of members of this phylum was generally predominant in the advanced stages of rock microbial colonization and specific studies indicated a key role of *Actinobacteria* in rock destructive and constructive processes and in the precipitation of secondary minerals^54–58^. Although some members of this phylum have also been described to have the capacity to fix nitrogen, *Actinobacteria* are mostly described for their heterotrophic metabolism and involvement in carbon turnover. Similarly to *Chloroflexi*, *Actinobacteria* are known to resist to desiccation, starvation and other stress conditions including rapid environmental changes (e.g., wet-dry and freeze–thaw cycling stresses)^13,59^. Further, *Chloroflexi* and *Actinobacteria* have cell wall properties typical of Gram-positive bacteria which were suggested to support the stable binding to silicate mineral surface, which might be involved in siliceous mineral nucleation processes^54^. In association with these features, these bacterial groups has been reported to co-colonize acidic and oligotrophic silica-based environments (e.g., Atacama deserts, dark volcanic ice cave systems located in Mt. Erebus)^9,11,13,14^.

The degradation activities of acidophilic *Actinobacteria* towards proteinaceous substrates was reported to release ammonia which induces the increase of the environmental pH. This aspect might find association with the alkalinisation process observed during the silica amorphization progression described in this work^60^. Further, *Actinobacteria* strains were suggested to be producers of pigmentation in coloured microbial mats present in different lava caves^61^. This consideration can find an association with the grey and black colour of the *Actinobacteria*-enriched speleothems SA and SC. In this study, specific actinobacterial genera were predominant in the amorphous silica speleothems and accounted for more than half of each microbial population, i.e., *Crossiella* in SA, *Acidothermus* and *Mycobacterium* in SC. Previous works reported the high abundance of *Pseudonocardiaceae* family (to which *Crossiella* genus belongs) in microbial mats from lava caves and weathered rocks from karst caves^56,62–64^. *Crossiella* was previously considered to have a key role in microbe-induced carbonate precipitation on stone monuments^65^. *Acidothermus* was detected in acidophilic and thermal environments^66^, including the cave walls of a thermal karst system^67^. *Mycobacterium* strains have been described to thrive in silica-rich environments (e.g., human lungs in silicosis), to colonize acidic environments and to be active in biomineralization processes due to specific enzymatic activities^68,69^. *Firmicutes Bacillus* and *Paenibacillus* were also found to be abundant genera in SA; however, they were only detected through one sequencing approach, probably due to primer utilization bias^70^. *Bacillus* spp. strains were widely studied for their biomineralization capacities associated with EPS production secretion and alkalinisation^71^. In particular, *Bacillus* strains were demonstrated to have silica solubilizing activities, which can support the role of these members in silica speleothem development^72,73^.

Previous studies reported the capacity of aerobic heterotrophic bacteria of the phyla *Chloroflexi*, *Actinobacteria* and *Acidobacteria* to utilize atmospheric trace gases as energy source under nutrient limiting conditions^19^. As the samples under analysis in this study are located in an oligotrophic setting and are dominated by members highly affiliated with trace gas oxidizers previously reported in other extreme and oligotrophic environments (i.e., lava tubes, desertic and polar sites)^18,59^, we characterized the diversity of *coxL* and *hypD* genes in QP, SA and SC that can be associated with bacterial trace gases oxidation activities. As a result, the majority of the sequences retrieved from these genes clone libraries were affiliated with those of the bacterial groups that were predominant in the microbial communities (based on the 16S rRNA gene analyses), i.e., *Chloroflexi*, *Acidobacteria* and *Actinobacteria*. In particular, dominant CoxL and HypD sequences from QP were affiliated with those from members of *Ktedonobacterales* and *Edaphobacter*, the latter being classified as an *Acidobacteria* genus. Interestingly, QP CoxL and HypD from *Chloroflexi* and *Acidobacteria* phyla were not distinctly distributed in the trees and the *Edaphobacter*-related sequences formed a separate clade from the acidobacterial sequences retrieved from the database. This is in line with 16S rRNA analysis, but also with possible horizontal gene transfer events, which frequently occur within *Chloroflexi* phylum in adverse environmental conditions^19,46^. In QP, the low abundant *Actinobacteria*, *Alphaproteobacteria* and *Verrucomicrobia* populations were shown to possess both the functional genes under analysis, being therefore possibly capable of CO and H_2_ oxidation during the first colonization of orthoquartzite surface. In this regard, previous transcriptional and functional studies indicated members of these phyla (e.g., *Bradyrhizobium* of *Alphaproteobacteria*) to utilize trace atmospheric gases as a way to adapt to oligotrophy and survive carbon limitation^21,74–76^. In the late stages of the coralloid speleothem formation, the possible metabolism of CO and H_2_ can be mainly attributed to *Actinobacteria* and, to a lower extent, to *Acidobacteria* and WPS-2 (*Candidatus* Eremiobacteraeota). These results are in line with the 16S rRNA analyses, being CoxL of *Pseudonocardiaceae* family and *Mycobacterium* genus predominant in the clone libraries of SA and SC, respectively. Accordingly, actinobacterial isolates, including *Mycobacterium smegmatis*, were found to oxidize atmospheric trace gases for survival during starvation conditions^21^. The metagenome-based reconstruction of a *Pseudonocardia* sp. genome from the Atacama Desert indicated the presence of pathways for H_2_, CO and C_1_ organic compounds utilization and CO_2_ fixation which might contribute to microbial development in the absence of direct photosynthetic inputs in extreme environments^59^.

In conclusion, this work describes with high taxonomic resolution the microbial communities colonizing three proximal portions of the floor surface in Imawarì Yeuta cave indicating a new bacterial lineage of *Ktedonobacterales* order as dominant in the first stage of orthoquartzite rock alteration and *Actinobacteria* members as featuring the subsequent silica amorphization stages ending up in a peculiar silica coralloid formation. The most abundant bacterial taxa within consecutive silica amorphization phases also possessed functional genes that were previously indicated to be involved in the oxidation of CO and H_2_ under nutrient limiting conditions and extreme environments^18–21^. The results presented in this work provide insights into the peculiar structure of microbial communities thriving in orthoquartzitic caves and into the bacterial taxa with the potential to utilize atmospheric trace gases as a possible metabolic strategy for microbial sustainment that needs to be further investigated in dark, oligotrophic, and silica-based environments.

## Materials and methods

### Sample collection

Samples were collected during a speleological expedition in March 2014. After scraping/collection with sterile tools, all samples were stored in eppendorf tubes filled with a solution of LifeGuard RNA solution. The transport from the site to the lab was carried out in a portable fridge, then samples were stored at − 80 °C until analysis.

### X-ray fluorescence spectrometry, X-ray diffraction and scanning electron microscopy

For X-Ray Fluorescence (XRF) spectrometric analyses, ultra-fine powered subsamples (~ 3 g) were embedded in rounded boric acid casts (~ 5 cm diameter, ~ 0.5 cm height) as previously described by Sauro et al.^10^. The XRF spectrometer (Axios-Panalytical) was equipped with a 4 kW Rh tube and SuperQ 3.0 software operating at the Department of Biological Geological and Environmental Sciences, University of Bologna^77^.

X-Ray Diffraction (XRD) analyses were conducted on 5 g sample by using a Philips PW3710 X-Ray diffractometer (current: 20 mA, voltage: 40 kV, range 2θ: 5°–80°, step size: 0.02° 2θ, time per step: 2 s) which mounted a Co-anode, as reported in Sauro et al.^10^. Data acquisition and processing were performed using the Philips High Score software package. All XRD operations were performed at the Department of Earth, Environment and Life (DISTAV), University of Genoa.

Scanning Electron Microscopy (SEM) and Field Emission Scanning Electron Microscopy (FESEM) analyses were conducted using a Vega3 Tescan and a Zeiss Supra 40 VP, respectively, operating at the DISTAV department and at the Department of Chemistry and Industrial Chemistry (University of Genoa, Italy). Before the analyses, samples required a gold sputtering-treatment. The sample preparation methods and the settings of both SEM and FESEM instruments were previously reported by Sauro et al.^10^.

### Total DNA extraction, Illumina MiSeq sequencing and EMIRGE

Total DNA was extracted from the three samples using the DNeasy Extraction Soil Kit (Qiagen) with slight modifications as previously described by Cappelletti et al.^78^. The DNA was quantified through QuBit (Thermofisher) before using it as template for 16S rRNA gene-targeting amplifications using different primer pairs listed in Table S1. Universal primer pairs 515F-907R^10^ were used to amplify the V4–V5 hypervariable region of 16S rRNA gene of both *Bacteria* and *Archaea* present in the samples. The PCR reactions targeting V4–V5 regions and corresponding Illumina sequencing were performed at the KAUST Genomic Core Lab (https://corelabs.kaust.edu.sa/) as previously described^10^. The reads were first trimmed for the indexes and primer sequences, and then checked for chimera and quality by using QIIME2 software. Reads were analysed using the DADA2 package version 1.5.0, as previously described in D’Angeli et al.^79^. Universal primer pairs 9F-1406R and 344F-1406R were respectively used to amplify the near full-length bacterial and archaeal 16S rRNA genes following PCR amplification procedures described by Koskinen et al.^80^. After shearing PCR amplicons using restriction endonucleases, libraries were prepared using NEB Next Ultra II FS DNA Library Prep Kit (New England Biolabs) according to the manufacturer protocol and using ten cycles of PCR. Paired-end Illumina sequencing was performed at the Core Facility Molecular Biology of the Medical University of Graz (Austria). Raw reads were trimmed and quality filtered^35^ using an in-house Galaxy set-up^81^, which included the algorithm Expectation Maximization Iterative Reconstruction of Genes from the Environment, referred to as EMIRGE, to carry out a probabilistic and reference-based reconstruction of full 16S rRNA genes from Illumina sequencing short-reads^35,36^. In particular, we utilized the script EMIRGE_amplicon.py that allows the reconstruction of complete 16S rRNA genes from PCR amplicon sequencing data^35^. Specifically, EMIRGE was run for 120 iterations with default parameters designed to merge reconstructed 16S rRNA genes if candidate consensus sequences share ≥ 97% sequence identity in any given iteration^35,36^. The starting candidate rRNA database was derived from SILVA SSU 132 reference database^82^. Reconstructed near-full length 16S rRNA sequences were clustered into Operational Taxonomic Units (named OTUs) at 97% identity to remove similar sequences. The reliability of 16S rRNA sequences reconstructed by EMIRGE is based on the fact that the algorithm handles possible PCR and sequencing errors by choosing the most-probable consensus for each 16S rRNA sequence based on the coverage acquired from multiple reads per consensus base^36^. Additionally, considering the number of reads that map to each reconstructed rRNA sequence, EMIRGE calculates abundance estimates for each full-length 16S rRNA gene^36^. OTUs with abundance ≥ 0.01% were proved to have a sufficient coverage to be considered reliable 16S rRNA sequences^35^. The probabilistic approach utilized by EMIRGE was proved to solve the problem encountered by the traditional assembly methods that were demonstrated to provide highly fragmented and misassembled 16S rRNA genes, due to the inability to solve the complexity arising from the co-assembly of highly homologous regions present in different 16S rRNA sequences^36^. Chimeras in both ASV and OTU datasets were identified and removed with Uchime2 v11^83^. Eukaryotic sequences were also excluded from further analysis. Taxonomic assignments were performed by using SILVA SSU 132 reference database^82^. The Illumina sequencing raw data were deposited in the Sequence Read Archive of NCBI under accession number PRJNA610757.

### Quantitative PCR

Quantitative PCR was conducted on the CFX96 Touch Real-Time PCR Detection System (Bio-Rad, Hercules, USA) by using SYBR green-based reactions. The quantification of the bacterial and archaeal 16S rRNA genes was performed in triplicate. The primer sets 338F-517R and 806F-945R^84^ were used to amplify the bacterial and the archaeal 16S rRNA genes, respectively (Table S1), in a 20 μL qPCR reaction mix containing: 10 ng total DNA, primers 300 nM each, 1× SsoAdvanced Universal SYBR Green Supermix (Bio-Rad, Hercules, USA), water (Lichrosolv; Merck, Darmstadt, Germany), using the thermocycling conditions: 95 °C for 15 min, 40 cycles of 94 °C for 15 s, 60 °C for 30 s, 72 °C for 40 s. Serial dilutions [across seven orders of magnitude (10^1^–10^7^)] of 16S rRNA gene PCR products from *Escherichia coli* and *Nitrososphaera viennensis* were used as standards for *Bacteria* and *Archaea*, as previously described^84^. The standard curves for *Escherichia coli* and *Nitrososphaera viennensis* showed correlation coefficients (R^2^) > 0.95 and qPCR efficiencies > 90% (Table S7).

### Clone libraries of 16S rRNA, *coxL* and *hypD* genes and screening through RFLP

Total DNA from all the three speleothems under analysis was used as template for PCR amplification reactions targeting the *coxL* and *hypD* genes using OmpF-O/Br^85^ and hypDfor2-hypDrev^86^ primer sets, respectively (Table S1). Total DNA extracted from QP was also used for the amplification of full-length 16S rRNA gene using 27F-1492R primers^76^ (Table S1). 16S rRNA clone library was only performed on QP sample as the Illumina sequencing analyses showed the dominance of novel bacterial lineages of *Ktedonobacterales* order that deserved further analysis in addition to reference-based 16S rRNA reconstruction methods (i.e., EMIRGE). For the three target genes, 10 ng of total DNA was added to a 50 µL (final volume) PCR reaction mixture containing Takara Ex Taq buffer with MgCl_2_ (10x; Takara Bio Inc., Tokyo, Japan), primers 200 nM each, dNTP mix 200 µM, Takara Ex Taq Polymerase 1.25 U. The amplification reactions of 16S rRNA gene were carried out under the following thermocycling conditions: 98 °C for 10 s, 30 cycles of 98 °C for 10 s, 55 °C for 30 s, 72 °C for 90 s, with a final extension at 72 °C for 20 min. The amplification reactions of *coxL* gene were carried out under the following thermocycling conditions: 98 °C for 10 s, 30 cycles of 98 °C for 10 s, 55 °C for 30 s, 72 °C for 90 s, with a final extension at 72 °C for 20 min. The amplification reactions of *hypD* gene were carried out under the following thermocycling conditions: 98 °C for 10 s, 30 cycles of 98 °C for 10 s, 55 °C for 30 s, 72 °C for 90 s, with a final extension at 72 °C for 20 min. After confirmation through electrophoresis, PCR products were purified with the Qiagen PCR Purification Kit (Qiagen, Hilden, Germany), ligated into the pCRII vector using the TOPO TA Cloning Kit (Invitrogen, San Diego, CA, USA), according to the manufacturer’s instructions, and finally cloned into *Escherichia coli* DH5α for clone library construction.

Restriction Fragment Length Polymorphism (RFLP) was performed on one hundred and forty library colonies of the 16S rRNA library and functional genes (*coxL* and *hypD*), respectively. Individual colonies were suspended in 20 μL of TE pH 8 and boiled for 5 min. After centrifugation for cell debris removal, 1 μL of the supernatant was used as template in PCR reactions and the amplicons were subject to enzymatic restriction for 3 h at 37 °C with 5 U of both *Alu*I and *Rsa*I enzymes, for the 16S rRNA gene colonies, and *Msp*I enzyme, for *coxL* and *hypD* gene colonies. Restriction profiles were analysed by running the restriction reactions on 2% (w/v) high resolution agarose gel electrophoresis with high-resolution agarose (Metaphor, Tebu-bio). Clones were clustered based on restriction patterns. RFLP screenings were stopped when the rarefaction curves approached saturation (coverage > 80%). For sequences identification, plasmids were purified from one representative clone from each cluster using the Qiagen Plasmid Purification Kit (Qiagen). Sequencing was performed by the Eurofins Genomics Service (Germany) using both T7 and T3 primers (Invitrogen) (Table S1). Chimeras were identified and removed with Uchime2 v11^83^. The clone library sequences were submitted to the NCBI Genbank database under accession numbers MT193405–MT193418 (16S rRNA sequences), MT193598–MT193613 (*coxL* sequences) and MT193585–MT193597 (*hypD* sequences).

### Phylogenetic and statistical analysis

Phylogenetic trees were constructed using (1) the sequences of the most abundant OTUs deriving from EMIRGE processing for all three samples (all these OTUs had abundance ≥ 0.01%, therefore possessing reliable reconstructed sequences due to enough sequence coverage^36^); (2) all the 16S rRNA gene sequences resulting from the clone library screening, only for the QP sample (due to the high abundance of ASVs and OTUs belonging to novel bacterial lineage within the *Ktedonobacterales* order, (3) the amino acid sequences of the CoxL and HypD obtained *from *in silico translation of the representative clone library clones that were sequenced, for all three samples (to detect the presence of functional genes possibly involved in atmospheric CO and H_2_ oxidation, respectively). For each sequence included in the tree, the most closely related sequences retrieved from the database (Best Blast Hits) were downloaded. All the sequences used for the phylogenetic analyses were aligned with Clustal Omega (https://www.ebi.ac.uk/Tools/msa/clustalo/) and used to construct a tree based on neighbour-joining clustering method using MEGAX^87^, with bootstrap values of 1000. Diversity indexes were calculated through Primer-E v7 (Primer-E Ltd, Plymouth, UK) on the basis of rarefied ASV and OTU datasets, filtered using a minimum relative abundance threshold of 0.002% and 0.01%, respectively^36,88^. Clustering analyses were performed on the basis of the presence and abundance of microbial genera by using Primer-E v7 and Bray–Curtis Distance Matrix.

## Supplementary information

Supplementary information.

## Data Availability

The authors confirm that the data supporting the findings of this study are available within the article and its supplementary materials.
