# Supplementary material for: Transition from unclassified Ktedonobacterales to Actinobacteria during amorphous silica precipitation in a quartzite cave environment
Source: Sci Rep. 2021 Feb 16;11:3921. doi: 10.1038/s41598-021-83416-5 (PMC7887251; doi:10.1038/s41598-021-83416-5)
Supplement: Supplementary file 1 — Supplementary information. [file 41598_2021_83416_MOESM1_ESM.docx]

Supplementary Material of

**Transition from unclassified *Ktedonobacterales* to *Actinobacteria* during amorphous silica precipitation in a quartzite cave environment**

Ghezzi D, Sauro F, Columbu A, Carbone C, Hong P-Y, Vergara F, De Waele J, Cappelletti M*

* Correspondence to: Martina Cappelletti, e-mail [martina.cappelletti2@unibo.it](mailto:martina.cappelletti2@unibo.it)

| **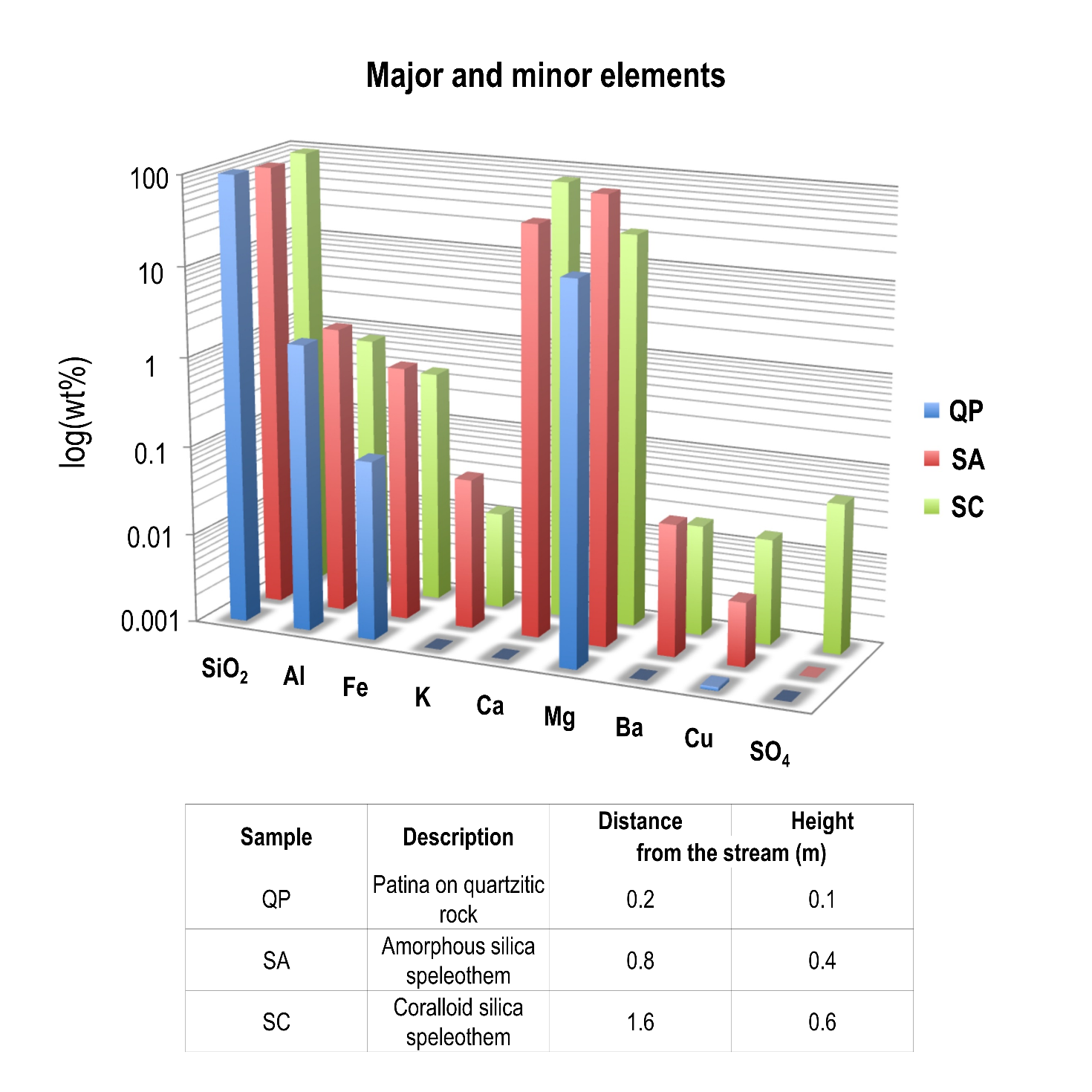** |
| --- |
| **Supplementary Figure S1.** Major and minor elements in log-wt% from XRF analysis (upper column graph) and relative position of the samples from the cave streambed (table below). |

| **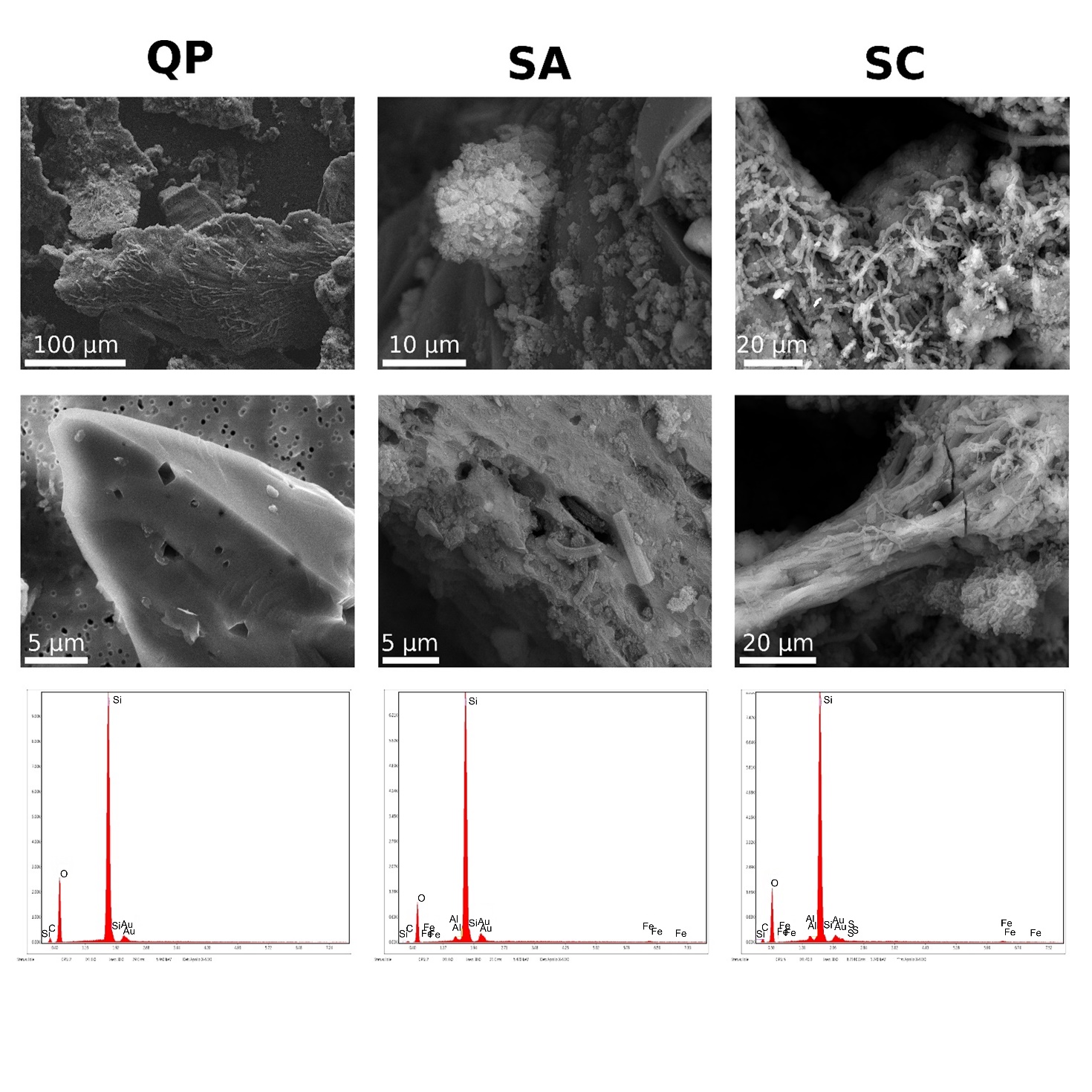** |
| --- |
| **Supplementary Figure S2.** FESEM images and EDS spectra of the three samples. QP) Fibrous biological structures (upper photo) covering pitted quartz grains (lower photo), with the EDS showing a predominant composition of silica dioxide and minor carbon. SA) Silicified biological structures (tubular hairy casts and flakes, respectively upper and lower photo), with the EDS showing a predominant composition of silica dioxide, minor carbon and iron. SC) Silicified clusters and filaments (respectively upper and lower photo), with the EDS showing a predominant composition of silica dioxide, minor carbon, iron and sulfur. |

| **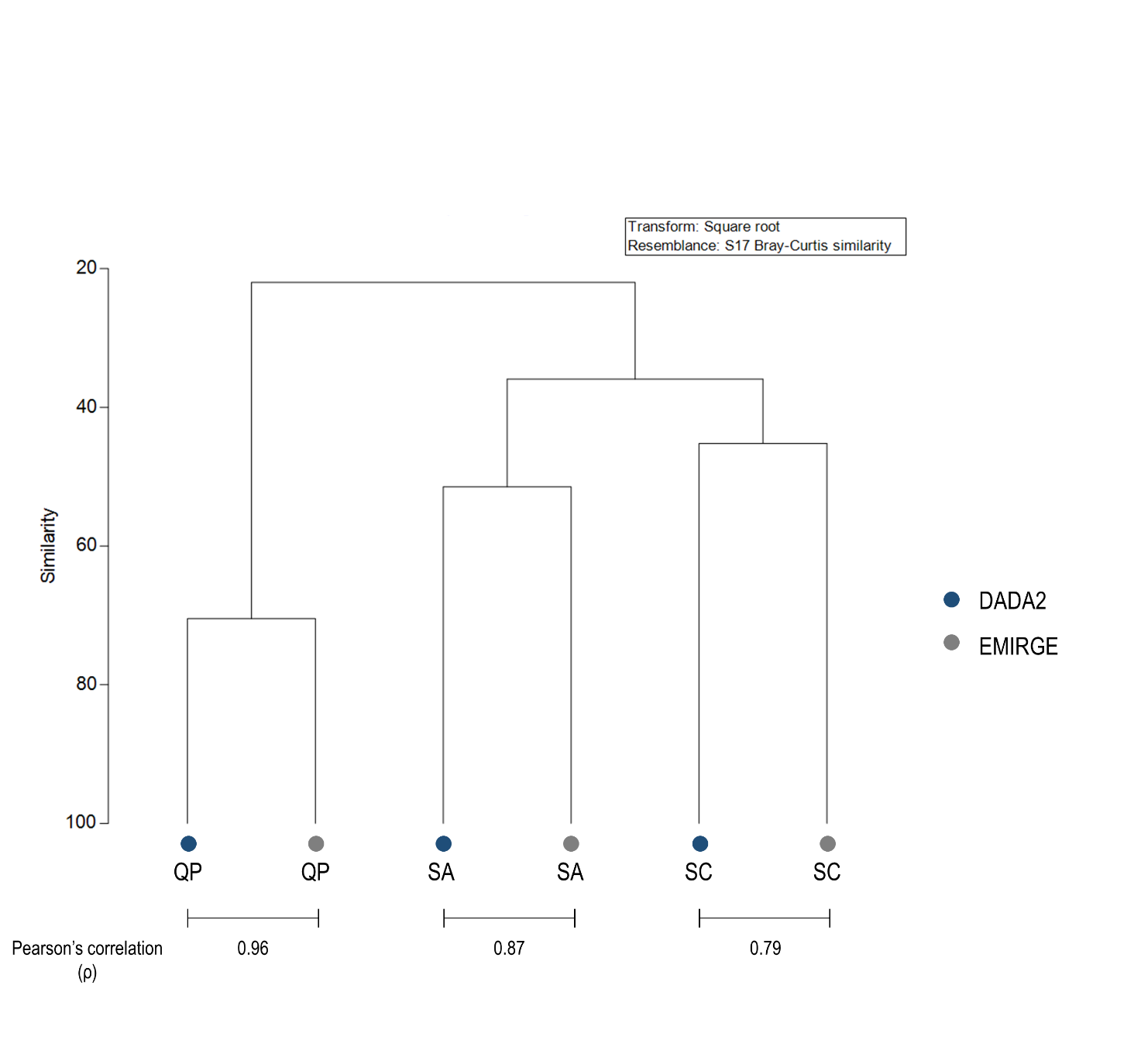** |
| --- |
| **Supplementary Figure S3.** Hierarchical cluster dendrogram of the microbial communities at genus level analyzed with DADA2 and EMIRGE based on Bray-Curtis similarity and Pearson’s correlation coefficient. |

| **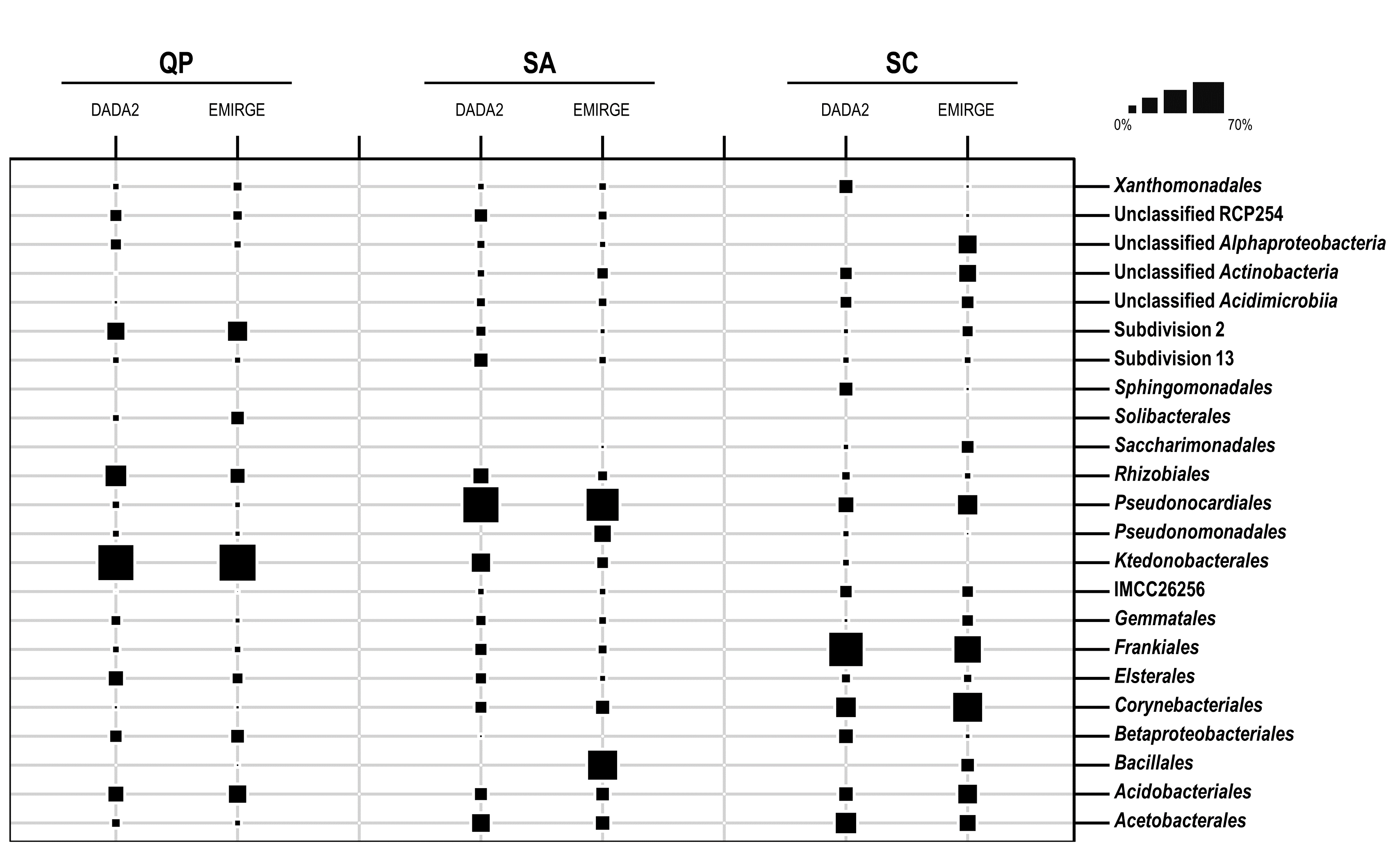** |
| --- |
| **Supplementary Figure S4.** Microbial community composition at order level with abundances of the three cave speleothems. Only orders with relative abundance > 1% in at least one sample are shown. |

| **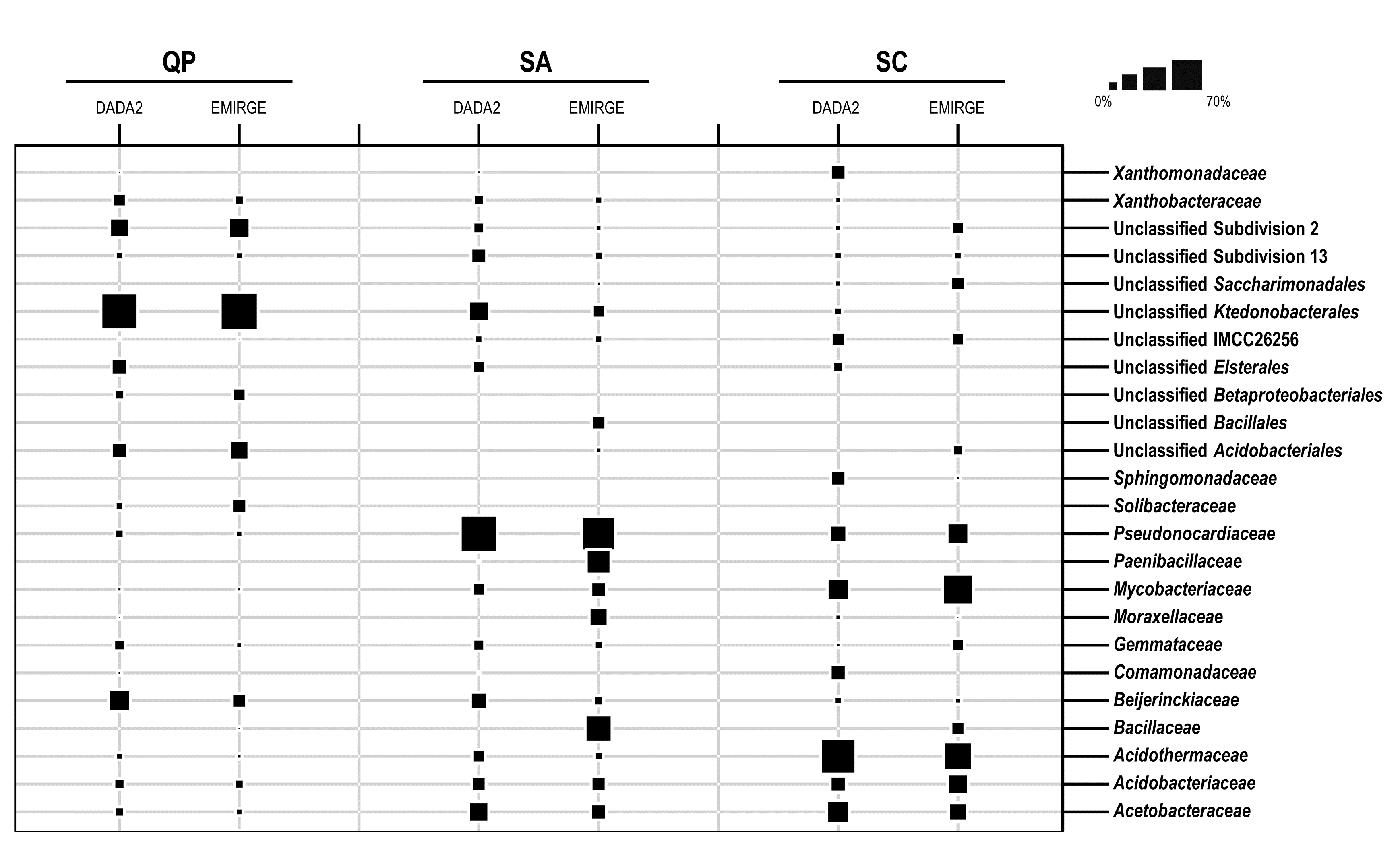** |
| --- |
| **Supplementary Figure S5.** Microbial community composition at family level of the three cave speleothems. Only families with abundance > 1% in at least one sample are shown. |

| **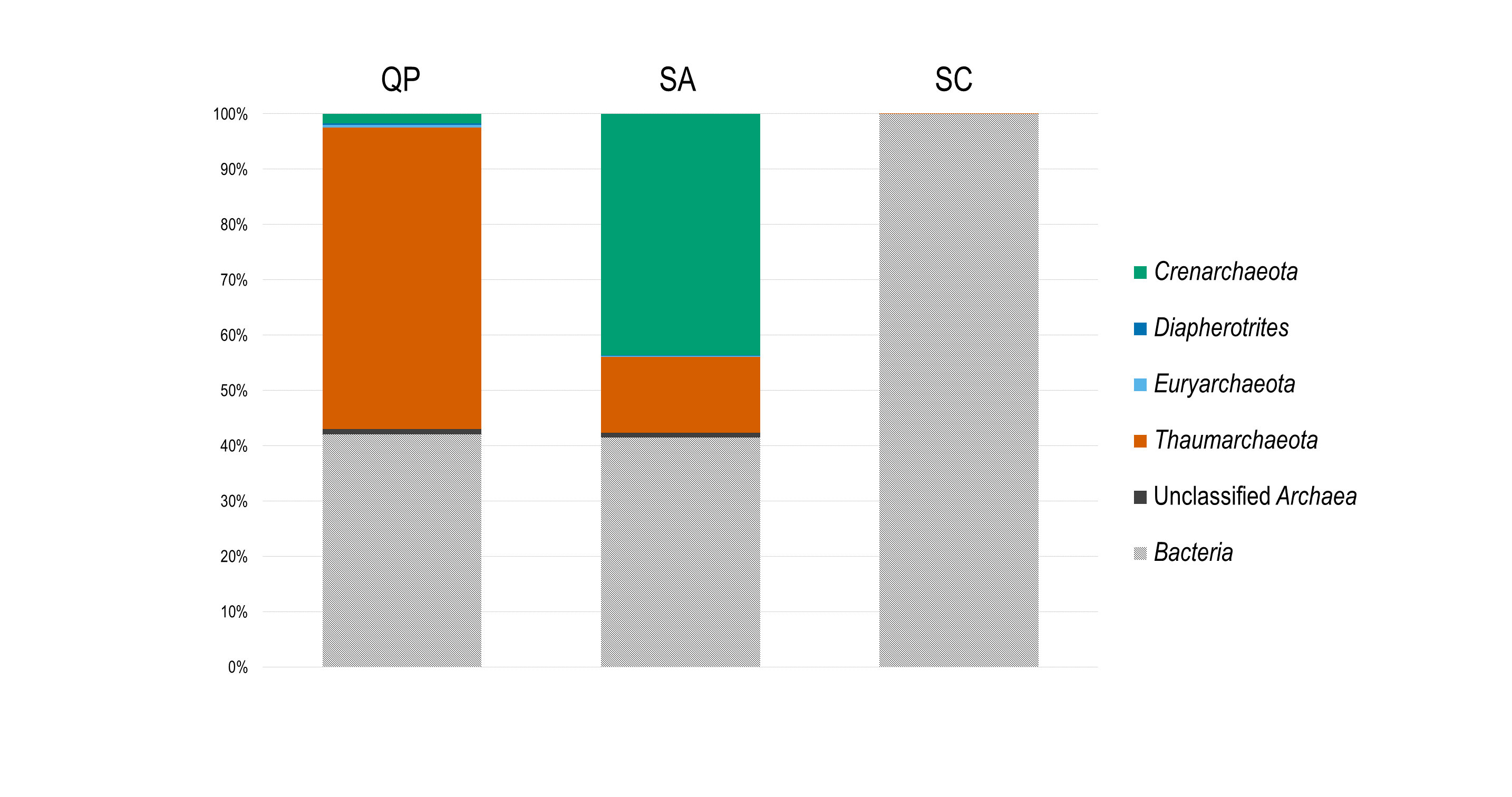** |
| --- |
| **Supplementary Figure S6.** Distribution of *Bacteria* and archaeal phyla in the three cave speleothems based on archaeal OTUs analysis. |

| **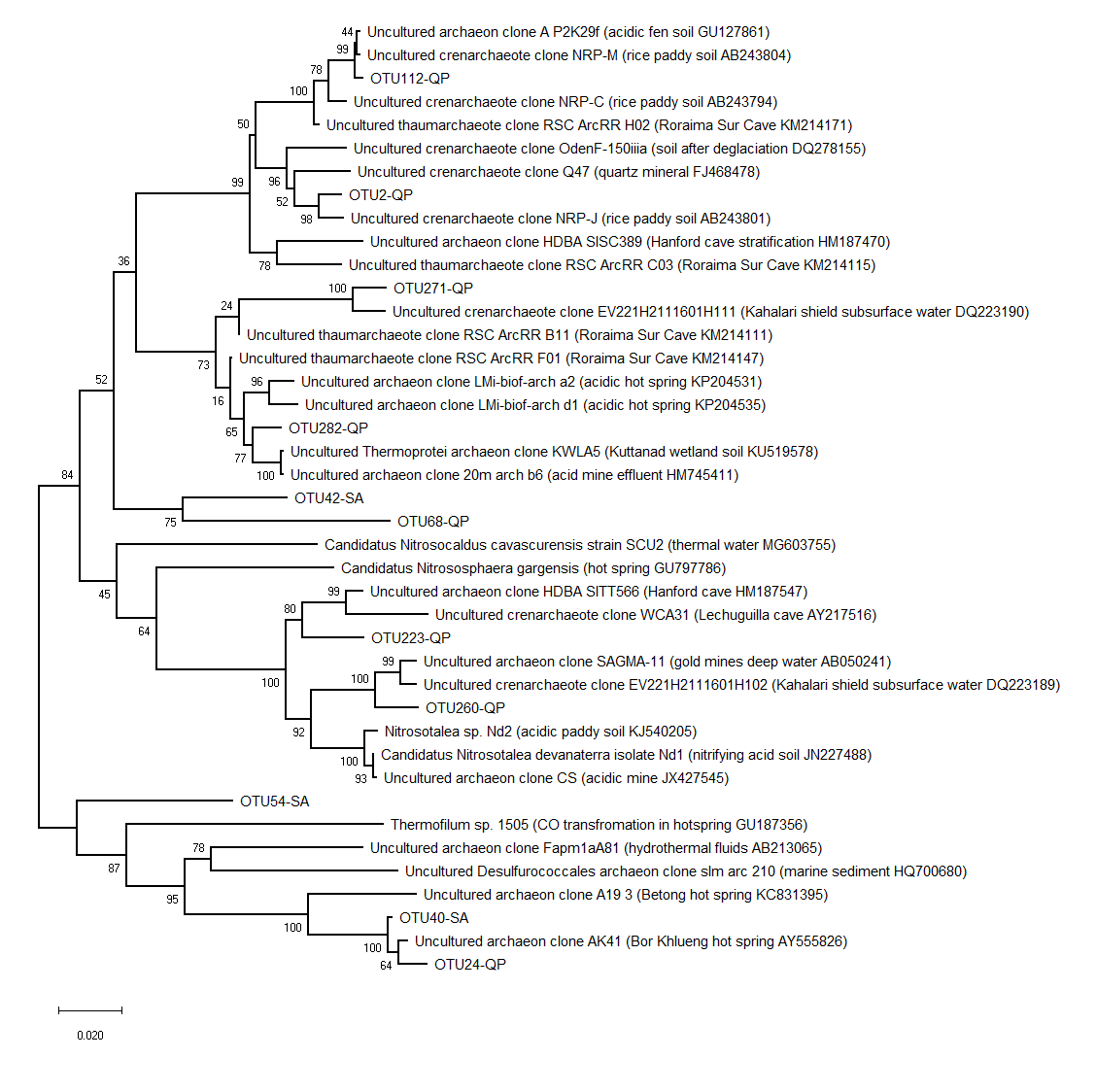** |
| --- |
| **Supplementary Figure S7.** Neighbor-joining tree showing the phylogenetic affiliation of the dominant archaeal OTUs in the three cave samples. |

**Table S1.** Primers used in the present study for PCR amplification and sequencing.

| **Method** | **Target genes** | **Samples** | **Primer set** | **Sequence (5’-3’)** |
| --- | --- | --- | --- | --- |
| qPCR | Bacterial  16S rRNA | QP, SA, SC | 338F | GCTGCCTCCCGTAGGAGT |
|  |  |  | 517R | ATTACCGCGGCTGCTGG |
| qPCR | Archaeal  16S rRNA | QP, SA, SC | 806F | GGACTACHVGGGTWTCTAAT |
|  |  |  | 945R | TAAAACTYAAAKGAATTGACGGG |
| V4-V5 sequencing | 16S rRNA | QP, SA, SC | 515F | GTGYCAGCMGCCGCGGTA |
|  |  |  | 907R | CCCCGYCAATTCMTTTRAGT |
| EMIRGE | Bacterial  16S rRNA | QP, SA, SC | 8F | AGAGTTTGATCCTGGCTCAG |
|  |  |  | 1100R | GGGTTGCGCTCGTTG |
| EMIRGE | Archaeal  16S rRNA | QP, SA, SC | 340F | CCCTAYGGGGYGCASCAG |
|  |  |  | 1000R | GGCCATGCACYWCYTCTC |
| Clone library | Bacterial  16S rRNA | QP | 27F | AGAGTTTGATCMTGGCTCAG |
|  |  |  | 1492R | TACGGYTACCTTGTTACGACTT |
| Clone library | *coxL* | QP, SA, SC | OmpF | GGCGGCTTYGGSAASAAGGT |
|  |  |  | O/Br | YTCGAYGATCATCGGRTTGA |
| Clone library | *hypD* | QP, SA, SC | hypD-for2 | GGNCCNGGCTGCCCGGTCTG |
|  |  |  | hypD-rev | GGCGNNGTGGTTTCAAANCC |
| Clone library | 16S rRNA*, coxL, hypD* | QP, SA, SC | T7 | TAATACGACTCACTATAGGG |
|  |  |  | T3 | ATTAACCCTCACTAAAGGGA |

**Table S2.** Summary of alpha diversity data for Illumina sequencing results.

| **Method** | **Speleothem** | **# reads** | **# ASVs** | **Shannon** | **Simpson’s** | **Evenness** |
| --- | --- | --- | --- | --- | --- | --- |
| DADA2 | QP | 177530 | 369 | 2.54 | 0.62 | 0.38 |
|  | SA | 58304 | 214 | 2.51 | 0.70 | 0.46 |
|  | SC | 185297 | 223 | 3.11 | 0.90 | 0.58 |
|  |  |  | **# OTUs** |  |  |  |
| EMIRGE | QP | 703467 | 773 | 3.31 | 0.81 | 0.50 |
|  | SA | 780587 | 443 | 4.15 | 0.94 | 0.68 |
|  | SC | 656922 | 332 | 4.38 | 0.97 | 0.76 |

**Table S3.** RFLP-based clone library analysis of QP sample and comparison with the OTU and ASV datasets.

| **Clone** | **% RFLP**  **dataset^a^** | **% OTU dataset**  **(# corresponding OTUs^b^)** | **% ASV dataset**  **(# corresponding ASVs^c^)** | **Taxonomy** |
| --- | --- | --- | --- | --- |
| QP-16S-clone10 | 68 | 41.0 (1) | 70.4 (2) | o_*Ktedonobacterales* |
| QP-16S-clone20 | 2 | 0 | 1.5 (4) | o_*Acidobacteriales* |
| QP-16S-clone24 | 1 | 1.1 (3) | 1.5 (4) | o_*Acidobacteriales* |
| QP-16S-clone26 | 5 | 1.1 (1) | 6.8 (10) | f_*Beijerinckiaceae* |
| QP-16S-clone27 | 2 | 0.3 (2) | 0.7 (4) | o_Subdivision 2 |
| QP-16S-clone59 | 10 | 1.1 (1) | 0.4 (4) | o_Subdivision 2 |
| QP-16S-clone62 | 4 | 0 | 70.4 (2) | o_*Ktedonobacterales* |
| QP-16S-clone63 | 1 | 0 | 0.001 (1) | f_*Chthoniobacteraceae* |
| QP-16S-clone66 | 1 | 0 | 0.005 (1) | f_*Phycisphaeraceae* |
| QP-16S-clone70 | 1 | 0 | 0.1 (2) | o_*Betaproteobacteriales* |
| QP-16S-clone73 | 1 | 11.1 (3) | 1.5 (8) | o_Subdivision 2 |
| QP-16S-clone74 | 2 | 0.001 (1) | 0.4 (4) | o_Subdivision 2 |
| QP-16S-clone96 | 1 | 0 | 70.4 (2) | o_*Ktedonobacterales* |
| QP-16S-clone99 | 1 | 3.5 (1) | 70.4 (2) | o_*Ktedonobacterales* |

a) % of the clones that were screened through Restriction Fragment Length Polymorphism (RFLP) in the clone library and that showed the same RFLP pattern of the representative clone that was Sanger sequenced b) “# corresponding OTUs” represent the number of OTUs sharing > 95% sequence similarity with the 16S rRNA clone; c) “# corresponding ASVs” represent the number of ASVs sharing > 97% sequence similarity with the 16S rRNA clone.

**Table S4.** Summary of alpha diversity data for clone libraries.

| **Target gene** | **Speleothem** | **# clones** | **# RFLP groups** | **Coverage** | **Shannon** | **Simpson’s** | **Chao1** |
| --- | --- | --- | --- | --- | --- | --- | --- |
| 16S rRNA | QP | 100 | 14 | 92 % | 0.61 | 0.54 | 22.0 |
| *coxL* | QP | 40 | 11 | 93 % | 0.92 | 0.86 | 12.0 |
|  | SA | 35 | 4 | 100 % | 0.57 | 0.72 | 4.0 |
|  | SC | 15 | 1 | 100 % | 0.0 | 0.0 | 1.0 |
| *hypD* | QP | 47 | 7 | 96 % | 0.45 | 0.46 | 47.3 |
|  | SA | 17 | 2 | 100 % | 0.29 | 0.48 | 2.0 |
|  | SC | 15 | 2 | 100 % | 0.26 | 0.40 | 2.0 |

**Table S5.** RFLP-based CoxL clone library analysis. For each clone, the most similar classified CoxL sequence retrieved from Genbank database is reported.

| **Sample** | **Clone** | **% library** | **Best Blast classified Hit** | | | |
| --- | --- | --- | --- | --- | --- | --- |
|  |  |  | **Taxonomy** | **Phylum** | **Identity (%)** | **Accession number** |
| QP | QP-CoxL-clone12 | 5 | *Edaphobacter aggregans* | *Acidobacteria* | 86 | WP_035348617 |
|  | QP-CoxL-clone15 | 2.5 | *Edaphobacter aggregans* | *Acidobacteria* | 85 | WP_035348617 |
|  | QP-CoxL-clone16 | 22.5 | *Ktedonobacter*  *sp.* | *Chloroflexi* | 91 | HAH00841.1 |
|  | QP-CoxL-clone21 | 5 | *Rhodospirillales bacterium* | *Proteobacteria* | 89 | OYV50419 |
|  | QP-CoxL-clone28 | 7.5 | *Rhodospirillales bacterium* | *Proteobacteria* | 84 | OYV50419 |
|  | QP-CoxL-clone35 | 2.5 | *Edaphobacter aggregans* | *Acidobacteria* | 88 | WP_035348617 |
|  | QP-CoxL-clone38 | 17.5 | *Bradyrhizobium oligotrophicum* | *Proteobacteria* | 97 | WP_015669260 |
|  | QP-CoxL-clone68 | 2.5 | *Ktedonobacter*  *sp.* | *Chloroflexi* | 94 | HAH00841.1 |
|  | QP-CoxL-clone76 | 20 | *Edaphobacter aggregans* | *Acidobacteria* | 84 | WP_035348617 |
|  | QP-CoxL-clone77 | 7.5 | *Edaphobacter aggregans* | *Acidobacteria* | 84 | WP_035348617 |
|  | QP-CoxL-clone81 | 7.5 | *Edaphobacter aggregans* | *Acidobacteria* | 86 | WP_035348617 |
| SA | SA-CoxL-clone5 | 11 | *Hydrogenophilales bacterium* | *Proteobacteria* | 85 | WP_147800236 |
|  | SA-CoxL-clone7 | 34 | *Pseudonocardia dioxanivorans* | *Actinobacteria* | 86 | WP_013674779 |
|  | SA-CoxL-clone79 | 29 | *Pseudonocardia dioxanivorans* | *Actinobacteria* | 85 | WP_013674779 |
|  | SA-CoxL-clone80 | 26 | *Pseudonocardia dioxanivorans* | *Actinobacteria* | 81 | WP_013674779 |
| SC | SC-CoxL-clone2 | 100 | *Mycobacterium interjectum* | *Actinobacteria* | 97 | WP_066917567 |

**Table S6.** RFLP-based HypD clone library analysis. For each clone, the most similar classified HypD sequence retrieved from Genbank database is reported.

| **Sample** | **Clone** | **% library** | **Best Blast classified Hit** | | | |
| --- | --- | --- | --- | --- | --- | --- |
|  |  |  | **Taxonomy** | **Phylum** | **Identity (%)** | **Accession number** |
| QP | QP-HypD-clone1 | 4 | *Edaphobacter aggregans* | *Acidobacteria* | 81 | WP_035352780 |
|  | QP-HypD-clone3 | 4 | *Edaphobacter aggregans* | *Acidobacteria* | 82 | WP_035352780 |
|  | QP-HypD-clone4 | 50 | *Edaphobacter aggregans* | *Acidobacteria* | 82 | WP_035358998 |
|  | QP-HypD-clone11 | 4 | *Pedosphaera parvula* | *Verrucomicrobia* | 93 | WP_007412928 |
|  | QP-HypD-clone16 | 11 | *Chloroflexi bacterium* | *Chloroflexi* | 94 | TME01503 |
|  | QP-HypD-clone28 | 10 | *Edaphobacter aggregans* | *Acidobacteria* | 81 | WP_035358998 |
|  | QP-HypD-clone29 | 1 | *Acidobacteria bacterium* | *Acidobacteria* | 82 | PYX55871 |
|  | QP-HypD-clone64 | 2 | *Acidobacteria bacterium* | *Acidobacteria* | 76 | OFV79518 |
|  | QP-HypD-clone72 | 14 | *Edaphobacter aggregans* | *Acidobacteria* | 82 | WP_035358998 |
| SA | SA-HypD-clone1 | 59 | *Candidatus* Eremiobacter | WPS-2 | 83 | PZR61729 |
|  | SA-HypD-clone12 | 41 | *Acidobacteria bacterium* | *Acidobacteria* | 88 | PYX55871 |
| SC | SC-HypD-clone14 | 60 | *Saccharomonospora viridis* | *Actinobacteria* | 91 | KHF45648 |
|  | SC-HypD-clone34 | 40 | *Candidatus* Eremiobacter | WPS-2 | 84 | PZR61729 |

**Table S7.** Correlation coefficients (R^2^), qPCR efficiencies and standard curve equations for quantification of *Bacteria* and *Archaea*.

| **Gene** | **R^2^** | **Efficiency** |
| --- | --- | --- |
| Bacterial  16S rRNA | 0.95 | 104% |
| Archaeal  16S rRNA | 0.98 | 91% |
